# TRIM21-mediated METTL3 degradation promotes PDAC ferroptosis and enhances the efficacy of Anti-PD-1 immunotherapy

**DOI:** 10.1038/s41419-025-07550-y

**Published:** 2025-04-03

**Authors:** Wenhao Mao, Qian Jiang, Yadan Feng, Chen Peng, Hui Peng, Xuan Li, Lin Jiao, Li Zhang, Liwei Ma, Ting Sun

**Affiliations:** 1 Department of Clinical Laboratory, The First Affiliated Hospital of Zhengzhou University, Zhengzhou, China; 2 Key Clinical Laboratory of Henan province, Zhengzhou, China; 3 Department of Oncology, The First Affiliated Hospital of Zhengzhou University, Zhengzhou, China; 4 State Key Laboratory of Oncology in South China, Guangdong Provincial Clinical Research Center for Cancer, Sun Yat-sen University Cancer Center, Guangzhou, China; 5 Department of Laboratory Medicine, West China Hospital, Sichuan University, Chengdu, China

**Keywords:** Oncogenes, Cancer therapy

## Abstract

Pancreatic cancer remains the most lethal human malignancy with limited clinical benefits from currently available anticancer treatments. Ferroptosis has recently attracted great attention as a potential antineoplastic strategy. However, the study of ferroptosis in PDAC remains insufficient. This study revealed that Methyltransferase like 3 (METTL3), as a key oncogenic factor, is frequently upregulated and inhibits ferroptosis by stabilizing SLC7A11 mRNA in PDAC. In addition, we identified a novel post-translational modification of METTL3 and characterized specific regulatory mechanisms of METTL3 protein degradation. The E3 ligase TRIM21 mediated K48-linked polyubiquitination of METTL3 at the K459 site, leading to the proteasomal degradation of METTL3, which prevented tumor progression by promoting ferroptosis. Interestingly, the TRIM21-METTL3 axics mediated ferroptosis effectively increased the expression of immune checkpoint PD-L1 and strengthened antitumor immunity in pancreatic cancer. Together, our findings first elucidated the detailed molecular mechanism of METTL3 degradation and revealed the pivotal role of the TRIM21-METTL3 axis in regulating ferroptosis and antitumor immunity, which may serve as a potential target for pancreatic cancer treatment.

## Introduction

Pancreatic cancer is a highly lethal malignancy characterized by difficulties in early diagnosis, chemoresistance, and a high incidence of comorbidities [1–5]. In current clinical practice, chemotherapy regimens have limited efficacy. Immunotherapy, which has shown promise in the treatment of other types of cancer, has generally failed to benefit pancreatic cancer patients [4, 6]. With pancreatic cancer projected to become the second-leading cause of cancer-related death in the next decade, new therapeutic strategies are urgently needed.

Emerging evidence suggests that N6-methyladenosine (m6A) RNA methylation is associated with diverse human diseases, especially cancer [7]. Multiple studies have demonstrated that methyltransferase like 3 (METTL3) mediated m6A modification can regulate oncogene expression and promote cancer progression through RNA processing, splicing, degradation, and translation [8–10]. METTL3 is significantly upregulated in pancreatic cancer tissues compared to paracancerous normal tissues, and its upregulation is highly associated with advanced pathological stages [11–13]. Moreover, it has been found that METTL3 can undergo diverse posttranslational modifications (PTMs), such as SUMOylation, lactylation and acetylation, which affect its m6A methyltransferase activity or subcellular localization [14–17]. However, the precise mechanisms by which METTL3 functions as an oncogene in pancreatic cancer and the upstream regulation of METTL3 degradation are largely require further investigation.

Currently, ferroptosis is recognized as a highly complex and strictly coordinated process that is mainly driven by iron accumulation and lipid peroxidation and controlled by integrated oxidation and antioxidant systems [18–21]. The accumulation of lethal amounts of reactive oxygen species (ROS) leads to DNA damage, genetic instability, and ultimately cell death. Accumulating evidence suggests that ferroptosis acts as an independent mechanism of tumor suppression [22, 23]. Therefore, ferroptosis induction has become a novel potential therapeutic strategy for cancer treatment. However, the study of ferroptosis in pancreatic cancer remains insufficient.

In this study, we revealed that TRIM21 induces the K48-linked polyubiquitination of METTL3 at the K459 site, leading to proteasome-mediated METTL3 degradation. Additionally, TRIM21-mediated METTL3 degradation induces ferroptosis by disrupting SLC7A11 mRNA stability and inhibits tumor progression during anti–PD-1 therapy. Our study elucidated the precise molecular mechanism of the TRIM21-METTL3 axis in pancreatic cancer and revealed that METTL3 may be a potential target in pancreatic cancer therapy.

## Materials and methods

### Cell culture and reagents

The cells utilized in this study were obtained from the American Type Culture Collection (ATCC, Manassas, VA), unless otherwise indicated. HPDE6C7 and AsPC-1 cells were cultured in RPMI-1640 medium (Gibco, NY, USA), while HEK293T, PANC-1, and MIA PaCa-2 cells were maintained in Dulbecco’s Modified Eagle Medium (Gibco, NY, USA). Additionally, MIA PaCa-2 cells required an additional 2.5% horse serum (Solarbio, Beijing, China) during the cultivation process. All culture media were supplemented with 10% fetal bovine serum (Gibco, NY, USA) and 1% penicillin/streptomycin antibiotic cocktail (Solarbio, Beijing, China) without mycoplasma in an incubator with 5% CO2 at 37 °C.

The other compounds used in this study were detailed in Table S1.

### shRNAs and plasmids construction

The shRNAs for METTL3 and TRIM21 were synthesized by GenePharma (Shanghai, China). The sense sequences (5’-3’) of shRNAs were showen in Table S2. HA-tagged ubiquitin (Ad. HA-ub) and other mutants, Flag-tagged METTL3 (Ad. Flag-METTL3), His-tagged TRIM21 (His-TRIM21) and various domain-deletion mutants, E3 ligase-dead mutant of His-TRIM21 (C16A) plasmid, lentiviral expression vectors were constructed by Fenghui Biotechnology Co., Ltd. (Hunan, China). The eleven lysine residues (K) mutants of Flag-METTL3 were constructed by Tsingke Biotechnology Co., Ltd. (Beijing, China).

### RNA isolation and real‑time quantitative PCR (RT‑qPCR)

Total RNA was extracted with RNAiso according to the manufacturer’s instructions. RNA purity and concentration were determined through the absorbance at A260/280 nm. DNase-treated RNA (1 μg) was used for cDNA synthesis using the cDNA one-step Synthesis kit (UE, Suzhou, China). RT-qPCR was performed in a QuantStudio system with the SYBR Premix ExTaq™ II (Takara, Japan). The RT-qPCR specific primers were synthesized by Tsingke Biotechnology Co., Ltd. (Beijing, China) and listed in Table S3. Experimental data were analyzed using the 2^−ΔΔCt^ method. GAPDH as an endogenous control to quantify the target genes expression.

### Western blotting and antibodies

Whole cells were lysed in pre-cooled RIPA lysis buffer (Beyotime, Shanghai, China) with a protease inhibitor cocktail (Selleck, Houston, USA). The same amount of total proteins was detected by sodium dodecyl sulfate-polyacrylamide gel electrophoresis (SDS-PAGE) according to the molecular weight of proteins. The isolated proteins were transferred to a polyvinylidine difluoride (PVDF) membrane (Millipore, Billerica, USA) and blocked with 5% skim milk for 2 h at room temperature. Subsequently, the membrane was reacted with specific primary antibodies overnight at 4 °C. Afterward, incubated with secondary antibodies conjugated with horseradish peroxidase 1 h at room temperature. Finally, immunoreactive proteins were detected with the Omni-ECL™ Femto Light Chemiluminescence Kit (Epizyme, Shanghai China). Antibodies used in this study were listed in Table S4.

### Immunofluorescence

For immunofluorescent staining, cells were fixed, permeabilized, blocked and incubated with primary antibodies overnight at 4 °C. Primary antibodies were used for reaction with tissue sections or cells overnight at 4 °C, followed by 1 h incubation with secondary antibodies at room temperature. Stained with DAPI to visualize the nuclei, the fluorescence was detected via confocal microscopy or electron microscope HITACHI Regulus 8100.

### Transmission electron microscopy

Briefly, cells were collected and immediately fixed in 3% phosphate-glutaraldehyde, treated with 1% osmium tetroxide, dehydrated, embedded, cut into ultrathin sections, and subsequently stained with uranyl acetate and lead citrate. The images were obtained using a transmission electron microscope.

### Co-inmunoprecipitation (Co-IP) and ubiquitination analysis

For co-IP, cell lysates in NP40 lysis buffer with protease inhibitor cocktail and the protein concentration of cell lysates were adjusted consistently through the BCA assay kit. Cell lysates were immunoprecipitated with the indicated antibodies overnight at 4 °C and then incubated with protein A/G Plus agarose (Santa Cruz Biotech, California, USA) mixed well for 2 h at 4 °C. The immunoprecipitated proteins were eluted and analyzed by immunoblotting.

In terms of ubiquitination analysis, the cells were co-transfected with target plasmid and HA-tagged ubiquitin (Ad. HA-Ub) and incubated in the presence of 10 µM MG132 (Selleck, Houston, USA) for 6 h. Experienced the process of co-ip experiment, the immunoprecipitated complexes were subjected to western blot analysis using ubiquitin antibody (Cell Signaling Technology, Boston, USA) or HA-Tag antibody (Abmart, Shanghai, China).

### Cell viability assays and cell death assays

Cell viability was assayed using Cell Counting Kit-8 (GlpBio, Montclair, USA) according to the instructions. Cell death was detected by flow cytometry using PI (Propidium Iodide) staining according to the instructions. The percentage of cell death was analyzed by Treestar FlowJo V10.6.2 software.

### GST-pull down

For GST pull-down, recombinant proteins GST-METTL3 and His-TRIM21 were constructed by inserting the coding regions of human genes METTL3 and TRIM21 into vectors pGEX-4T-1 and pET-28a, which were induced in *E. coli* BL21 and grown in LB-ampicillin with IPTG (Sangon, Shanghai, China) at 16 °C overnight. Fusion proteins His-TRIM21 and GST-METTL3 were purified and incubated with shaking overnight at 4 °C. Pull-down samples were captured by glutathione-sepharose beads, immunoblotting was used to analyze the bound proteins.

### RNA immunoprecipitation (RIP) assay and RIP-seq

RIP assay was conducted using a Magna RIP Kit (Millipore, New Bedford, MA) with the guidance of manufacturer’s protocols. Briefly, cells were collected and lysed in RIP lysis buffer, followed by centrifugation, the supernatant was incubated with Protein-A/G agarose beads and antibodies at 4 °C overnight. The extracted immunoprecipitated RNAs were purified and analyzed by RT‑qPCR.

The extracted immunoprecipitated-RNAs were measured and analyzed by RT‑qPCR. For RNA sequencing, immunoprecipitated-RNAs were sequenced at aksomics (shanghai, China) using the DNBSEQ-T7 platform. When the screening factor fold change was 1.0, too many genes were included in the analysis, so the threshold for comparison was increased to more than 1.2. Further data analysis based on gene expression levels, such as GO function significance analysis, pathway significance analysis and other data analysis were performed through company’s custom program python/R/shell.

### Methylated RNA immune‑precipitation (MeRIP)

Total RNA from pancreatic cancer cells was extracted using TRIzol reagent and treated with DNase to eliminate genomic DNA. The cleaved RNA fragments were incubated with 5 μg anti-m6A specific antibody (Synaptic Systems, Germany) or anti-IgG for 2 h at 4 °C. Then, the mixture was incubated with protein G beads (Invitrogen, California, USA) overnight at 4 °C. After proteinase K buffer digestion, the eluted RNA fragments were extracted and concentrated by RNA Clean & Concentrator^TM^-25 kit (zymo research, Orange County, USA). Finally, the relative expression of RNA was detected with qPCR and normalized by the input samples.

### mRNA stability measurement and protein stability assay

Briefly, pancreatic cancer cells were treated according to our experimental design. Samples were treated with 5 μg/mL actinomycin D (MedChemExpress, New Jersey, USA) for 0, 4, 8 h. At these indicated times, total RNA was extracted and mRNA levels were quantified by qRT-PCR.

To assess the impact of TRIM21 on METTL3 protein degradation, pancreatic cancer cells were transfected for 48 h and then treated with 100 μg/ml cycloheximide (GlpBio, Montclair, USA) for 0, 3, 6, 9, 12 and 15 h. The protein stability of METTL3 was determined using western blotting.

### Lipid peroxidation assay, intracellular ROS, and GSH/GSSG ratio measurement

C11-BODIPY581/591 (DOJINDO, Japan) was used for the lipid peroxidation assay in cells with different treatments. Cells were incubated with C11-BODIPY (5μΜ) in serum-free medium and measured by Flow Cytometer FACS (FITC, 484 nm/510 nm). Meanwhile, the concentration of intracellular malondialdehyde (MDA) was examined using lipid peroxidation MDA assay kit (Beyotime, Shanghai, China). Cells were lysed in pre-cooled lysis buffer and measured the protein concentration. The sample proteins were reacted with thiobarbituric acid (TBA) and the levels of MDA were evaluated by microplate reader with the absorbance at 532 nm.

In addition, intracellular ROS levels were detected by the peroxide-sensitive fluorescent probe 2’, 7’-dichlorofluorescein diacetate (DCFH-DA, Beyotime, Shanghai, China). Cells were incubated with 10 μM DCFH-DA and nuclei were stained with Hoechst. The mean fluorescence intensity (MFI) of the cells was observed under a fluorescence microscope or flow cytometer with FITC channel (484 nm/ 510 nm). The ratio of glutathione to oxidized glutathione (GSH/GSSG Ratio) in cells was measured through the GSH/GSSG Ratio Detection Assay Kit (Beyotime, Shanghai, China).

### Immune profiling by flow cytometry

Fresh tumors were digested and filtered into a single-cell suspension through a 70 µm strainer. Red Blood Cell Lysis Buffer was used to lyse the erythrocytes. The samples were blocked with anti-mouse CD16/32 to prevent non-specific binding. Surface staining (anti-CD3, anti-CD45, anti-CD4, anti-CD8) was conducted according to instructions. For the assessment of intracellular cytokines, cells were stimulated with Cell Activation Cocktail (BioLegend, California, USA) and resuspended in Fixation/ Permeabilization solution (eBioscience, California, USA). Cells were stained with anti-IFNγ and Granzyme B before profiling by flow cytometry. All samples were analyzed with FlowJo software and the relative antibodies were listed in Table S4.

### Animal experiment

The animal experiments were approved by the Animal Protection Committee of Zhengzhou University (No. ZZU-LAC20230331 [04]). The mice were purchased from Gempharmatech Co., Ltd and fed in the Animal Center affiliated with Zhengzhou University under the specific-pathogen-free (SPF) conditions.

BALB/c nude mice were randomly divided into 6 groups and five mice per group to ensure the stability of the results. For the subcutaneous tumor models, 2 × 10^6^ MIA PaCa-2 cells with different lentivirus treatments were injected subcutaneously into 5-week-old BALB/c nude mice. When the tumor grew to 50 mm^3^, mice were treated daily with vehicle or ferrostatin-1 (S7243, Selleckchem) (20 mg/kg) in 0.1% DMSO, 2.5% PEG300,0.25% Tween80 for 2 weeks.

To assess the impact of TRIM21-mediated METTL3 on pancreatic cancer immunotherapy. 5 × 10^5^ PANC-02 cells with different lentivirus treatments were subcutaneously injected into the flank of C57BL/6 mice. After tumors were established, 100 µg anti-PD-1 antibody was intraperitoneally injected on the indicated days.

Tumor volumes were measured with a vernier caliper every 2 days and calculated by the following formula: volume (cm^3^) = (short diameter)^2^ × (long diameter)/2 [24]. The mice were sacrificed by cervical dislocation and the tumor tissues were taken out for further experiments including measured, weighed, and histologic analyzed.

### Pancreatic cancer tissue and immunohistochemistry

In total, 90 pairs pancreatic cancer tissues and paired para-cancer tissues were prepared by Shanghai Outdo Biotech Co., Ltd. In 90 patients, two patients could not be evaluated the protein expression of METTL3 due to tissue damage in the pathological sections; one patient died perioperatively, and the pathological stage of one patient was not detailed. The baseline characteristics of tissues were shown in Table S5.

For immunohistochemistry, tissues were fixed and embedded with paraffin blocks. After the process of de-paraffinization, dehydration, antigen extraction and blocking, the paraffin sections were incubated with primary antibodies at 4 °C overnight and the immunohistochemical staining was visualized with horseradish peroxidase conjugates diaminobenzidine using Diaminobenzidine detection. Sections were counterstained with hematoxylin and observed under light microscopy. Immunoreactivity score (IRS) was used to evaluate immunoreactivity [25]. IRS = staining intensity (SI)×positive percentage (PP).

### Kaplan-Meier survival analysis

Kaplan-Meier survival analysis curves (log-rank test, *P* values displayed) were generated using Rstudio. Excluding deaths occurred during perioperative period, cancer-specific survival was measured from 14 days after surgery until the date of death in pancreatic cancer. Patients were excluded in cases where survival data were lacking or pathology slides were not accessible.

### Statistical analysis

All experiments were repeated at least three times. Data were analyzed by appropriate statistical using GraphPad Prism 8 (GraphPad Software Inc. USA) and shown as mean ± standard deviation. Differences with *p*-value < 0.05 were considered statistically significant. (*P < 0.05, **P < 0.01, ***P < 0.001, ****P < 0.0001).

## Results

### METTL3 is upregulated in pancreatic cancer

**Fig. 1:**
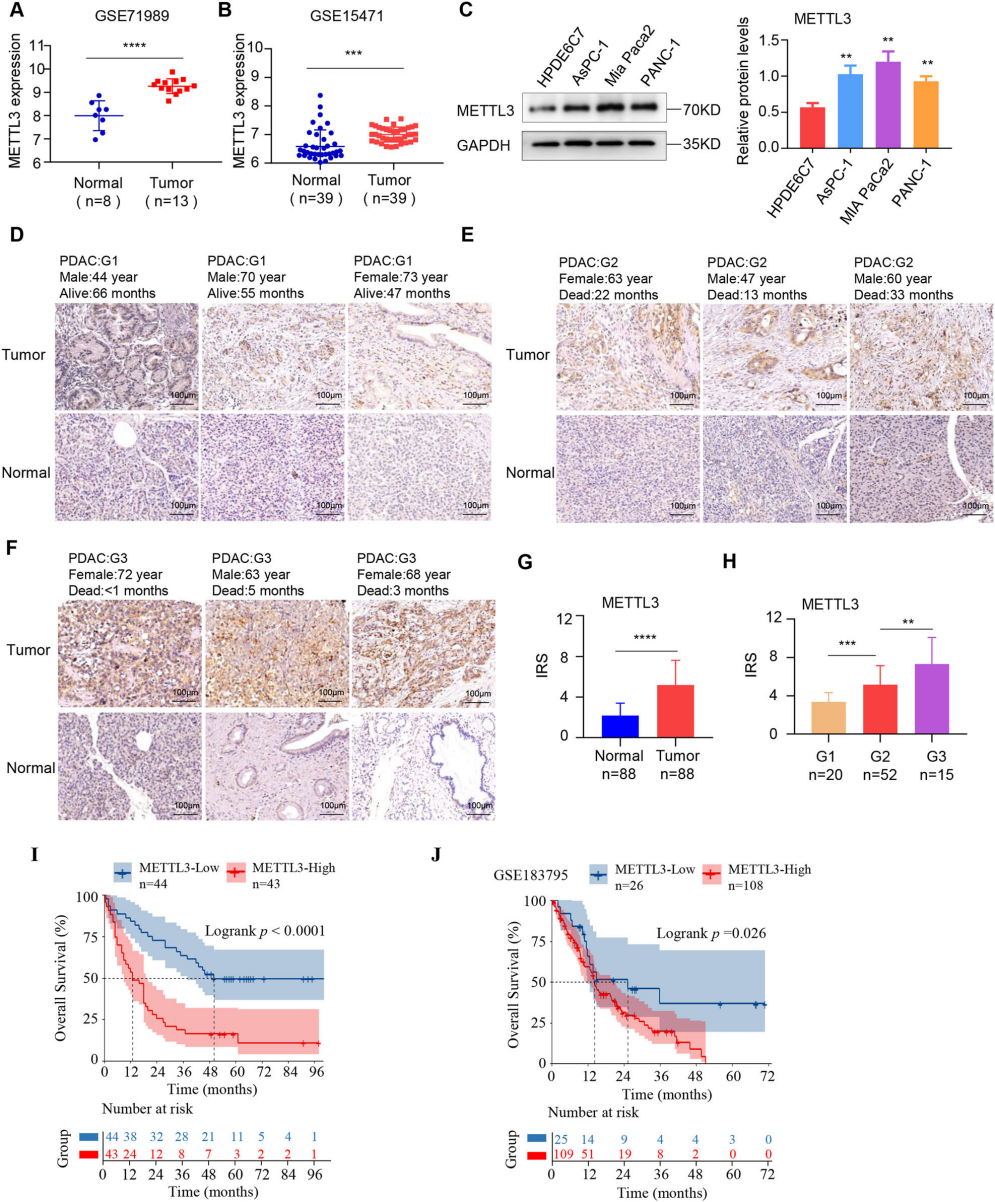
METTL3 is upregulated in pancreatic cancer. **A**, **B** METTL3 expression between pancreatic cancer tissues and normal tissues in GEO database. **C** The protein expression of METTL3 in cell lines was detected by western blotting. **D**–**F** Representative images of METTL3 IHC staining in paired pancreatic cancer and adjacent normal tissues with different malignancy histologic grades (G1–G3). Scale bars, 100 μm. **G** The degree of METTL3 immunohistochemical staining was evaluated in pancreatic cancer and adjacent normal tissues according to the immunoreactive score (IRS). **H** IRS of METTL3 IHC staining in tumors with different malignancy histological grades (G1–G3). **I** Kaplan–Meier survival plots (log-rank test, *P* values displayed) demonstrated the prognostic impact of METTL3 expression in pancreatic cancer. **J** Kaplan–Meier survival plots exhibited the effect of METTL3 expression on the prognosis of pancreatic cancer in GEO database GSE183795. (*P < 0.05, **P < 0.01, ***P < 0.001, ****P < 0.0001).

Previous studies have reported that m6A modifications play crucial roles in pancreatic cancer [26, 27]. In this study, we investigated the expression of m6A regulators in pancreatic cancer tissues and normal tissues based on the GSE71989 and GSE15471 GEO datasets. Our analysis revealed an overall trend of increased m6A regulator expression in pancreatic cancer (Supplementary Fig. S1A-B). Consistently, METTL3 expression was significantly elevated in pancreatic cancer tissues (Fig. 1A, B). Furthermore, we analyzed the protein expression of METTL3 in pancreatic cancer cell lines. Compared to the human pancreatic ductal epithelial cell line HPDE6C7, METTL3 expression was notably upregulated in multiple pancreatic cancer cell lines (Fig. 1C).

Additionally, apart from tissue damage in the pathological sections, we finally examined METTL3 protein levels in 88 pairs tissues by immunohistochemistry analysis, and the results revealed increased METTL3-positive staining in pancreatic cancer tissues compared to adjacent normal tissues (Fig. 1D–G). In addition, higher METTL3 protein expression was found to be positively correlated with histological grade (G1-G3), as shown in Fig. 1H. Kaplan-Meier survival analysis showed that the median survival time of patients with higher METTL3 expression was shorter than that of patients with lower METTL3 expression (Fig. 1I). Similar survival results were also observed in the GSE183795 GEO database, which contains prognostic data for pancreatic cancer patients (Fig. 1J).

In summary, these results indicated that METTL3 was significantly upregulated and positively correlated with histological grade and poor prognosis in pancreatic cancer.

### TRIM21 directly interacts with METTL3

To investigate the regulatory mechanisms underlying METTL3 upregulation in pancreatic cancer, we isolated METTL3-associated protein complexes from pancreatic cancer cells by co-IP assay. The extracted METTL3-associated proteins were then analyzed by LC‒MS/MS. As shown in Supplementary Fig. S2A, the Venn diagram demonstrates the overlap of three independent LC‒MS/MS analyses, and 122 proteins were identified as common highly reliable METTL3-interacting proteins. Mass spectrometric analysis revealed six unique peptide sequences that matched TRIM21, which is an E3 ubiquitin ligase belonging to the tripartite motif (TRIM)-containing protein family [28] (Supplementary Fig. S2B and Supplementary Fig. S2C-H). Furthermore, the analysis also identified two ubiquitin protein peptides (Supplementary Fig. S2I-J). Previous research demonstrated that TRIM family proteins promote the degradation of their substrates through the ubiquitin-proteasome pathway [29]. Thus, it is plausible that TRIM21 may interact with METTL3 and facilitate the connection between ubiquitin and METTL3.

**Fig. 2:**
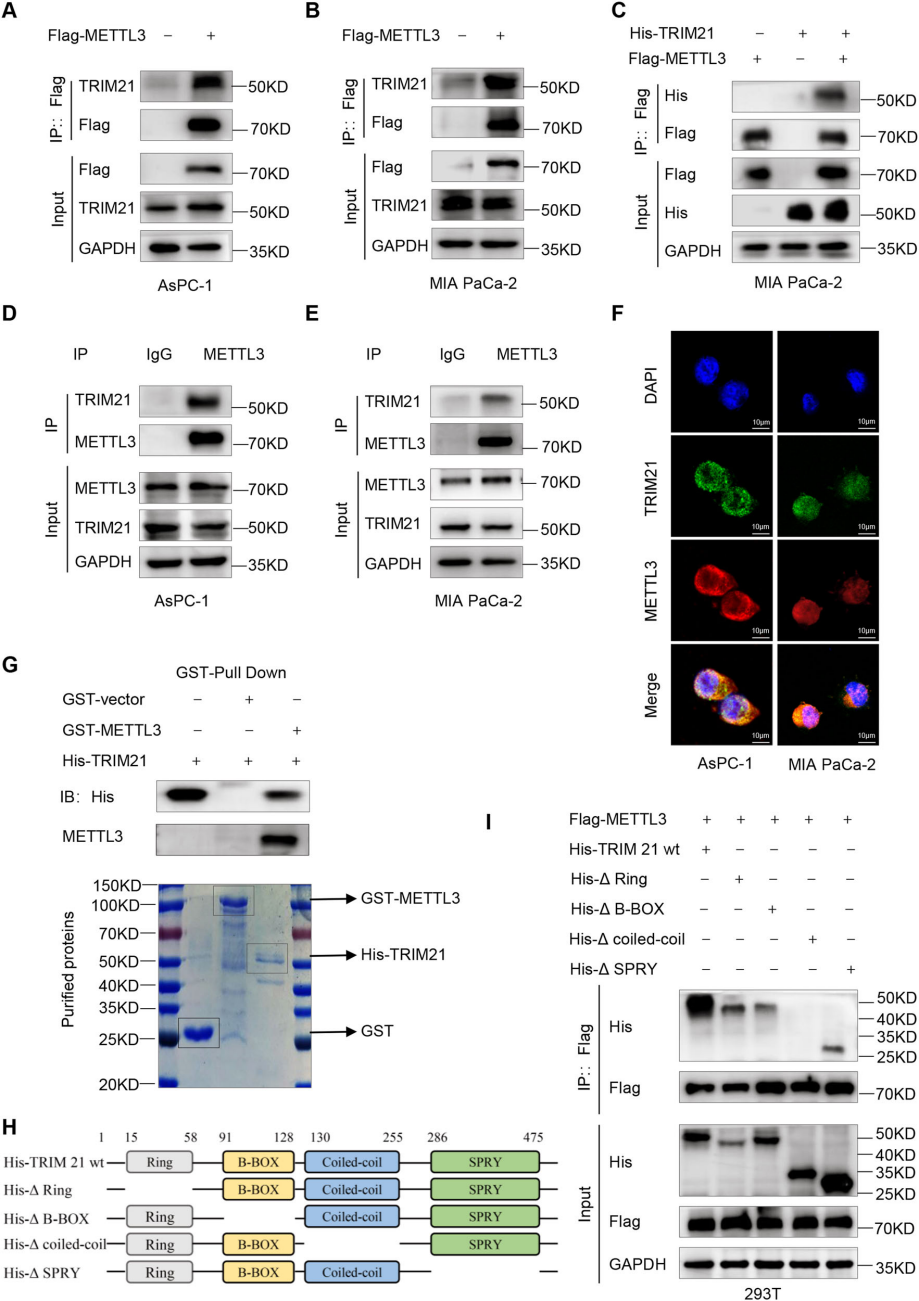
TRIM21 directly interacts with METTL3. **A**, **B** Flag-tagged METTL3 was transfected into AsPC-1 and MIA PaCa-2 cells for 48 h, followed by immunoprecipitation with the anti-Flag antibody and immunoblotting analysis with anti-TRIM21 antibody. **C** Immunoprecipitation analysis with anti-Flag antibody was used to detect the interaction between exogenous Flag-METTL3 and His-TRIM21 in MIA PaCa-2 cells. **D**, **E** Immunoprecipitation with anti-METTL3 antibody verified the interaction between endogenous METTL3 and TRIM21 in pancreatic cancer cells. **F** Confocal immunofluorescence of endogenous METTL3 colocalized with TRIM21 stained by anti-METTL3 (red) and anti-TRIM21 (green) antibodies in pancreatic cancer cells. Scale bars, 10 μm. **G** Western Blot analysis for pulldown of purified His-TRIM21 incubated with purified GST or GST-METTL3 fusion protein. **H** Schematic of TRIM21 and different domain-deletion mutants. **I** Immunoprecipitation analysis with anti-Flag antibody to explore the interaction domain of TRIM21.

To validate the interaction between METTL3 and TRIM21, we transfected pancreatic cancer cells with a Flag-tagged METTL3 expression vector or a vector encoding His-tagged TRIM21. As shown in Fig. 2A, B, TRIM21 was readily detected in the complexes that were immunoprecipitated with Flag-METTL3. Conversely, METTL3 was coimmunoprecipitated with His-tagged TRIM21 in a similar fashion (Supplementary Fig. S3A-B). Furthermore, the simultaneous expression of the two exogenous proteins in cells further confirmed the interaction between Flag-METTL3 and His-TRIM21 (Fig. 2C and Supplementary Fig. S3C). To evaluate this interaction under more physiological conditions, we performed co-IP assays with endogenous proteins in human pancreatic cancer cells. As shown in Fig. 2D, E, the endogenous TRIM21 protein was coprecipitated by a METTL3-specific antibody, while endogenous METTL3 was coprecipitated by a TRIM21-specific antibody (Supplementary Fig. S3D-E). The colocalization results were consistent with the coimmunoprecipitation data (Fig. 2F). To determine whether TRIM21 and METTL3 interact directly, GST pull-down assays shown in Fig. 2G, TRIM21 strongly bound to immobilized GST-METTL3 but not GST alone. These data demonstrate that TRIM21 is a bona fide binding partner of METTL3 both in vitro and in vivo.

TRIM21 is a member of the TRIM family, and it consists of multiple domains, including a RING domain with E3 ubiquitin ligase activity at the N-terminus, a B-box domain, a coiled-coil domain, and a SPRY domain at the C- terminus [30, 31]. We generated a series of TRIM21 domain-deletion mutants to examine which domain is responsible for its interaction with METTL3 (Fig. 2H). As shown in Fig. 2I, deletion of the coiled-coil domain abolished the association between TRIM21 and METTL3, indicating that these proteins interact via the coiled-coil domain of TRIM21.

### TRIM21 promotes the proteasomal degradation of METTL3

**Fig. 3:**
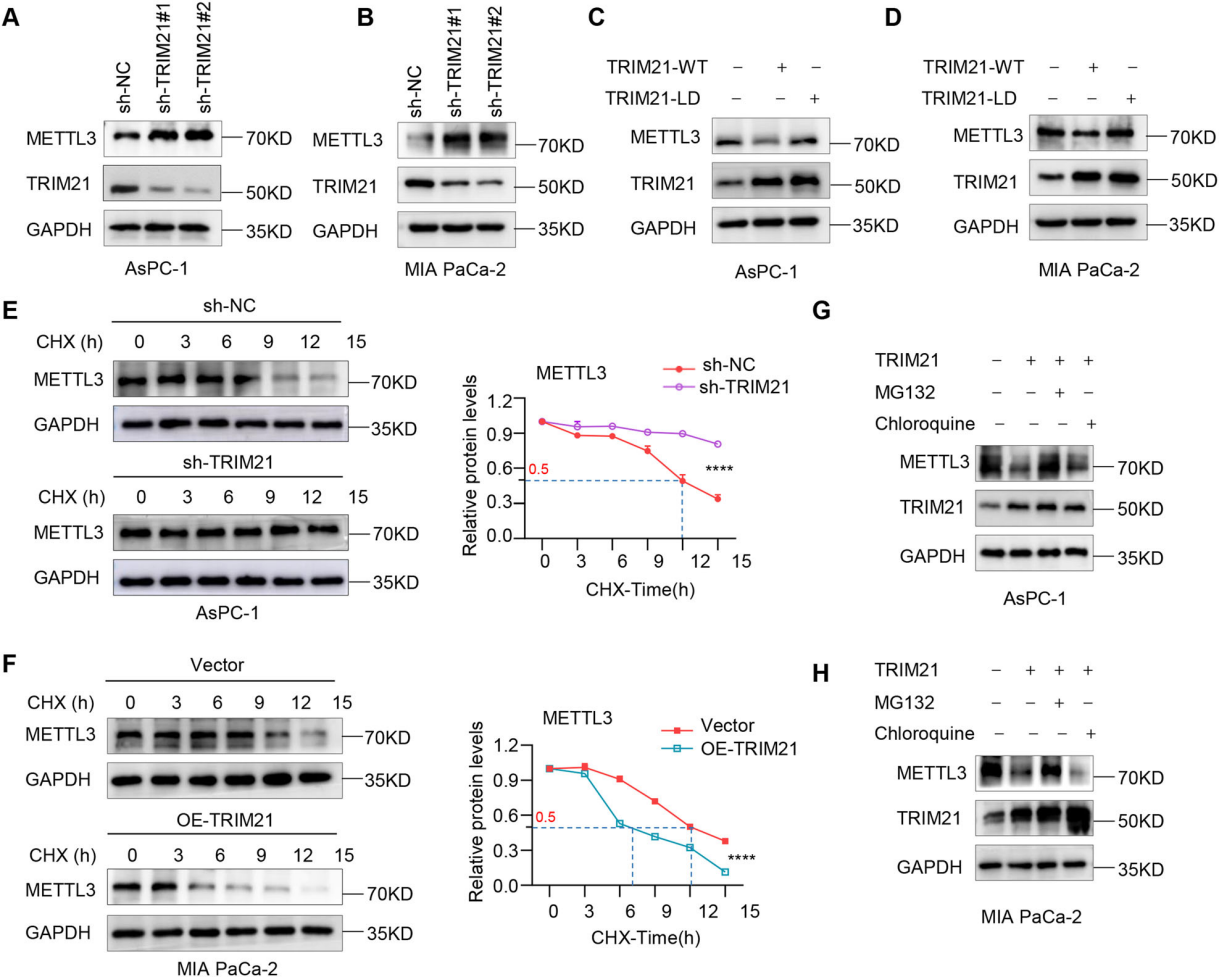
TRIM21 promotes the proteasomal degradation of METTL3. **A**, **B** The protein expression levels of METTL3 were detected by western blot assays in AsPC-1 and MIA PaCa-2 cells transfected with sh-TRIM21. **C**, **D** The protein expression levels of METTL3 were detected by western blot assays in AsPC-1 and MIA PaCa-2 cells transfected with TRIM21-WT or TRIM21-LD mutant. **E**, **F** In the presence of CHX (100 μg/ml), cell proteins were detected at the indicated time to analyze the content of METTL3 protein in the lysate. **G**, **H** Western blot analysis for METTL3 protein expression in TRIM21 overexpression cells followed by the treatment with MG132 (10 μM) or chloroquine (10 μM) for 6 h. (*P < 0.05, **P < 0.01, ***P < 0.001, ****P < 0.0001, ns not significant).

To understand the functional consequences of this interaction, we first examined whether the expression of TRIM21 affects the METTL3 protein levels in pancreatic cancer cells. Overexpression of TRIM21 was associated with decreased protein levels of METTL3 (Supplementary Fig. S4A-B), whereas depletion of TRIM21 dramatically elevated the METTL3 protein levels (Fig. 3A, B). In particular, there were no obvious differences in METTL3 mRNA expression after TRIM21 knockdown or overexpression (Supplementary Fig. S4C-F).

Simultaneously, to elucidate whether the regulation of METTL3 occurs due to the E3 ubiquitin ligase activity of TRIM21, we generated an E3 ligase-defective TRIM21 mutant by mutating cysteine 16 to alanine (C16A, denoted TRIM21-LD) [32]. Overexpression of TRIM21-WT significantly decreased METTL3 expression, whereas TRIM21-LD had no effect (Fig. 3C, D, supplementary Fig. S4G-H). These results indicated that TRIM21-mediated degradation of METTL3 is dependent on E3 ligase activity.

In addition, cycloheximide (CHX) chase assays were used to investigate the kinetics of the effect of TRIM21 on METTL3 stability. TRIM21 knockdown inhibited METTL3 protein degradation in CHX-treated pancreatic cells in Fig. 3E. Moreover, the half-life of the METTL3 protein was significantly reduced by the overexpression of TRIM21-WT (Fig. 3F), but not by the overexpression of TRIM21-LD (Supplementary Fig. S4I). Furthermore, the proteasome inhibitor MG132, but not the lysosome inhibitor chloroquine, reversed the TRIM21-induced decrease in the METTL3 protein levels (Fig. 3G, H). Taken together, these results indicated that TRIM21 promotes METTL3 degradation via the proteasomal pathway.

### TRIM21 mediates the K48-linked polyubiquitination of METTL3 at K459 site

**Fig. 4:**
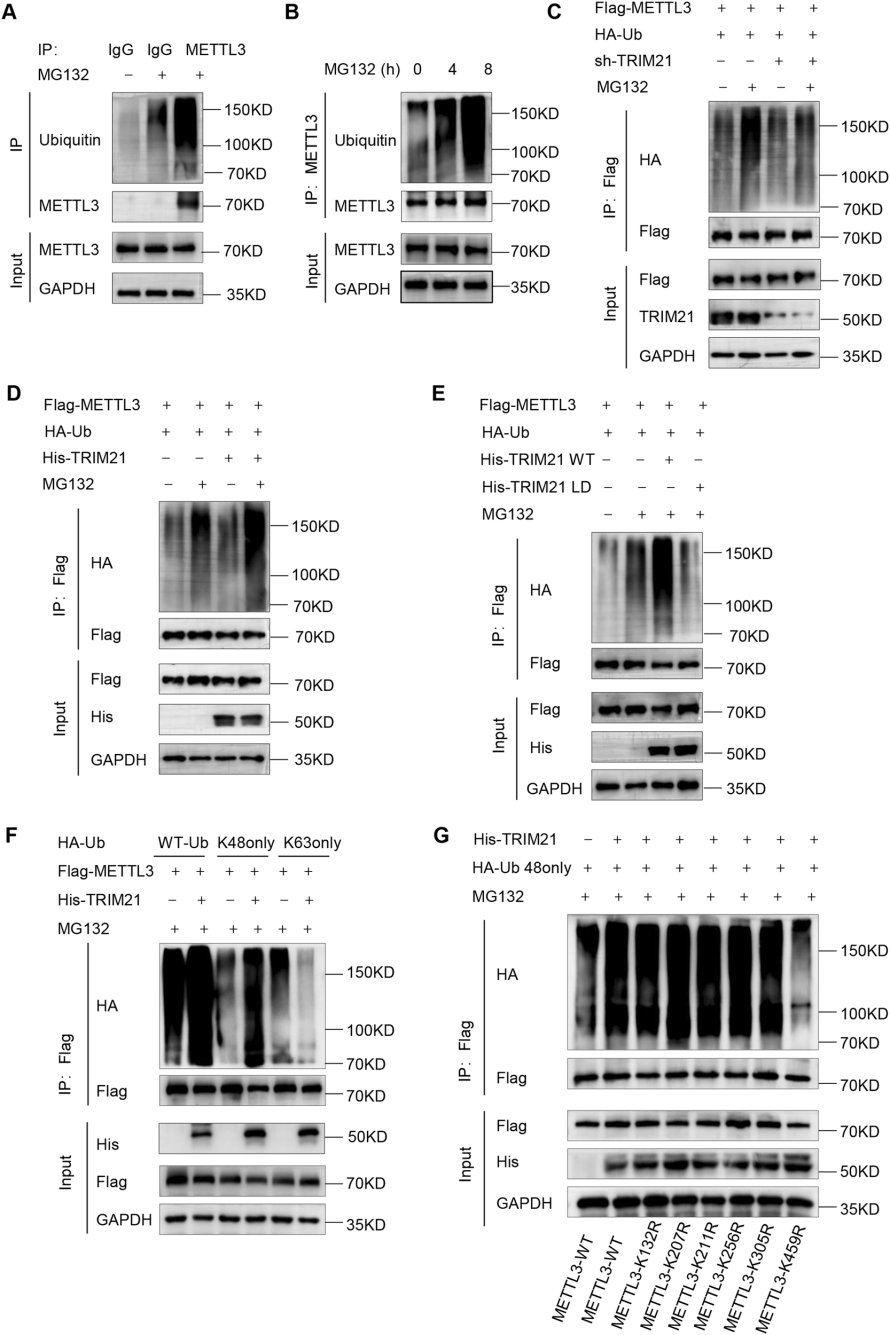
TRIM21 mediates the K48-linked polyubiquitination of METTL3 at K459 site. **A** IP-IB of ubiquitinated METTL3 in AsPC-1 cells treated with MG132 (10 μM) for 8 h. **B** IP-IB of ubiquitinated METTL3 in AsPC-1 cells treated with MG132 for different time. **C** IP-IB of ubiquitinated METTL3 using AsPC-1 cells transfected with Flag-METTL3, HA-ubiquitin and/or sh-TRIM21, followed by treatment with MG132. **D** IP-IB of ubiquitinated METTL3 using MIA PaCa-2 cells transfected with Flag-METTL3, HA-ubiquitin and/or His-TRIM21, followed by treatment with MG132. **E** IP-IB of ubiquitinated METTL3 using MIA PaCa-2 cells transfected with Flag-METTL3, HA-ubiquitin and/or His-TRIM21 WT or His-TRIM21-LD, followed by treatment with MG132. **F** IP-IB of ubiquitinated METTL3 using 293 T cells transfected with Flag-METTL3, HA-Ub (WT, K48 only or K63 only) and/or His-TRIM21 and treated with MG132. **G** Flag-METTL3 or arginine substitution mutants were expressed in 293 T cells and Lysates were prepared for IP-IB.

Considering that TRIM21 functions as an E3 ligase, we analyzed the polyubiquitination of endogenous METTL3, which was further enhanced in pancreatic cancer cells treated with the proteasome inhibitor MG132 (Fig. 4A, B). To test the hypothesis that TRIM21 mediates the ubiquitination of METTL3, we transfected Flag-tagged METTL3 and HA-tagged ubiquitin into HEK 293 T cells. As expected, knockdown of TRIM21 significantly reduced the ubiquitination of METTL3 (Supplementary Fig. S5A). In contrast, TRIM21 overexpression markedly increased the ubiquitination of METTL3 after MG132 treatment (Supplementary Fig. S5B). However, the ligase defective mutant TRIM21 (C16A) failed to mediate the polyubiquitination of METTL3 (Supplementary Figure S5C). Meanwhile, we found the same results in pancreatic cancer cells (Fig. 4C–E), indicating that the E3 ligase activity of TRIM21 is required for METTL3 ubiquitylation.

K48 and K63-linked polyubiquitin chains are the two most abundant types of polyubiquitin chains, involved in the ubiquitin-proteasome degradation pathway and cell signaling pathways, respectively [33, 34]. We observed that K48-only ubiquitin, but not K63-only ubiquitin, led to TRIM21-mediated METTL3 ubiquitylation (Fig. 4F). This is consistent with the mass spectrometry results shown in Supplementary Fig. S2I-J. The ubiquitin protein has a ubiquitinated modification at K48, which means that the ubiquitin that interacted with METTL3 was K48-linked polyubiquitination. Further verification was conducted by changing the K63 and K48 sites of ubiquitin to arginine (R). K48R, but not K63R ubiquitin, blocked TRIM21-mediated METTL3 ubiquitination (Supplementary Fig. S5D).

To gain deeper insights into the molecular mechanisms underlying the ubiquitination of METTL3, we generated arginine substitution mutants at each of the eleven predicted lysine sites in METTL3, as identified by the online tool (http://plmd.biocuckoo.org/), namely K132R, K207R, K211R, K256R, K305R, K459R, K480R, K488R, K513R, K576R, and K578R. Additionally, we generated double mutants at adjacent lysines, such as K207R/K211R, K480R/K488R, and K576R/K578R in the METTL3 protein. Our findings demonstrated that all the mutants, except for K459R, had no significant effect on TRIM21-mediated METTL3 ubiquitination (Fig. 4G and Supplementary Fig. S5E-F). Sequence analysis of the METTL3 protein revealed that K459 is a relatively conserved site among various species (Supplementary Fig. S5G). Therefore, our results suggested that TRIM21 mediates the K48-linked polyubiquitination of METTL3 at the K459 site.

### TRIM21 promotes ferroptosis by targeting METTL3 in pancreatic cancer

**Fig. 5:**
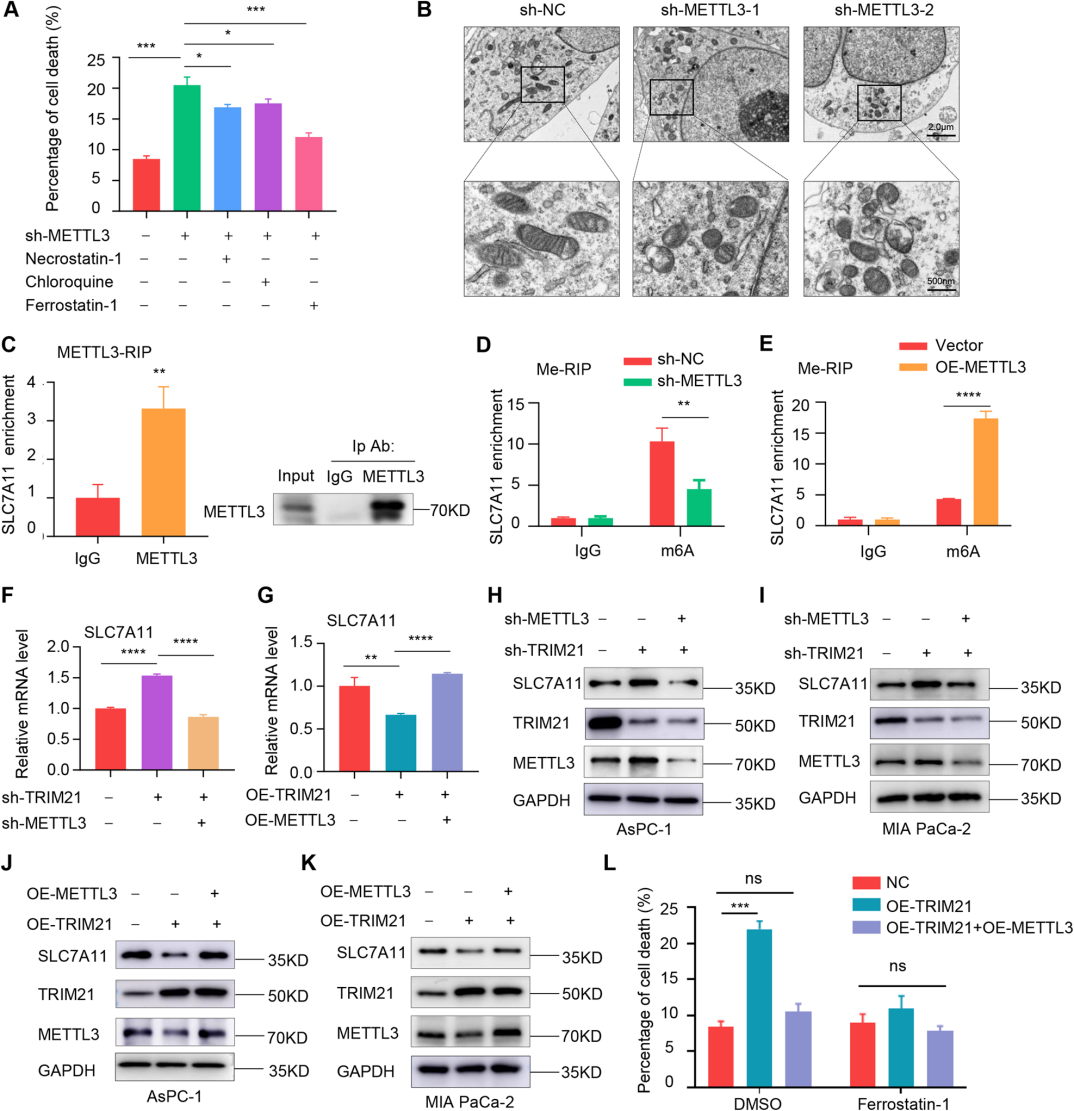
TRIM21 promotes ferroptosis by targeting METTL3 in vitro. **A** Flow cytometry analysis was used to determine cell death rates in subgroups of METTL3-knockdown cells treated with various cell death inhibitors. **B** The electron microscopy images show changes in mitochondria following treatment with shMETTL3 in MIA PaCa-2 cells. Scale bar, 2.0μm and 500 nm. **C** RIP-qPCR revealed the binding enrichment of SLC7A11 to METTL3 in MIA PaCa-2 cells. Western blot was used to verify the immunoprecipitation efficiency of METTL3 protein. **D**, **E** MeRIP-qPCR analysis demonstrated the impact of METTL3 on mediating m6A methylation modification of SLC7A11 mRNA in pancreatic cancer cells. **F**, **G** The SLC7A11 mRNA expression was measured by RT-qPCR in pancreatic cancer cells with the intervention of TRIM21 and METTL3. **H–K** The Altered protein expression of SLC7A11 was measured by western blot in subgroups. **L** The cell death induced by TRIM21 overexpression was effectively rescued by METTL3 overexpression or treatment with ferroptosis inhibitors ferrostatin-1 (1 μM). (*P < 0.05, **P < 0.01, ***P < 0.001, ****P < 0.0001, ns not significant).

In the experimental process, we observed that knocking down METTL3 expression promoted cell death (Supplementary Fig. S6A-B). To further explore which pathway is involved in METTL3-mediated cell death. The results showed that knockdown of METTL3 increased the proportion of dead cells, while treatment with ferrostatin-1 significantly reversed this effect. In contrast, inhibitors of necroptosis (necrostatin-1) and autophagy (chloroquine) had only minor effects (Fig. 5A). And ferrostatin-1 could restore cell viability reduced by METTL3 knockdown (Supplementary Fig. S6C). Morphologically, METTL3 deletion induced ferroptosis-specific changes of mitochondria (Fig. 5B), including volume reduction, loss of mitochondrial cristae, and increased bilayer membrane density, consistent with previous reports [18, 35]. RNA immunoprecipitation sequencing (RIP-seq) was used to explore the targets of METTL3 in regulating ferroptosis. In supplementary Fig. S6D-E, the Gene Ontology (GO) analysis and Kyoto Encyclopedia of Genes and Genomes (KEGG) pathway enrichment revealed that METTL3-related RNA targets were closely associated with the process of ferroptosis. Gene set enrichment analysis (GSEA) further revealed significant enrichment of SLC7A11 mRNA with METTL3 in regulating the glutathione metabolic process (Supplementary Fig. S6F-G). This finding was validated by RIP-qPCR in MIA PaCa-2 cells (Fig. 5C). Then, methylated RNA immunoprecipitation (meRIP)-qPCR analysis showed that METTL3 was involved in mediating the m6A methylation of SLC7A11 mRNA (Fig. 5D, E). Therefore, SLC7A11 was selected as a potential target of METTL3 in pancreatic cancer, and the role of SLC7A11 in the ferroptosis pathway is illustrated in Supplementary Fig. S6H. Furthermore, both the protein and mRNA levels of SLC7A11 were positively correlated with METTL3 expression (Supplementary Fig. S7A-F). For mechanism, RNA decay assay indicated that METTL3 regulates SLC7A11 expression by increasing mRNA stability (Supplementary Fig. S7G-H). In pancreatic cancer cells, METTL3 regulated ferroptosis and lipid peroxidation levels through SLC7A11 (Supplementary Fig. S8A-F).

We further investigated whether TRIM21 is involved in the induction of ferroptosis via the METTL3/SLC7A11 axis. Our results showed that TRIM21 negatively regulates the expression of SLC7A11 at both the mRNA and protein levels, and these effects could be reversed by METTL3 (Figs. 5F, G and H–K). Additionally, METTL3 expression remarkably reduced the cell death in TRIM21-overexpression cells to a similar degree as Ferrostatin-1 treatment (Fig. 5L). Consistent with these findings, METTL3 expression or Ferrostatin-1 reversed the alterations in GSH/GSSG ratio, cellular lipid peroxidation, and ROS levels induced by TRIM21 overexpression in pancreatic cancer cells (Supplementary Figure S9A-D).

**Fig. 6:**
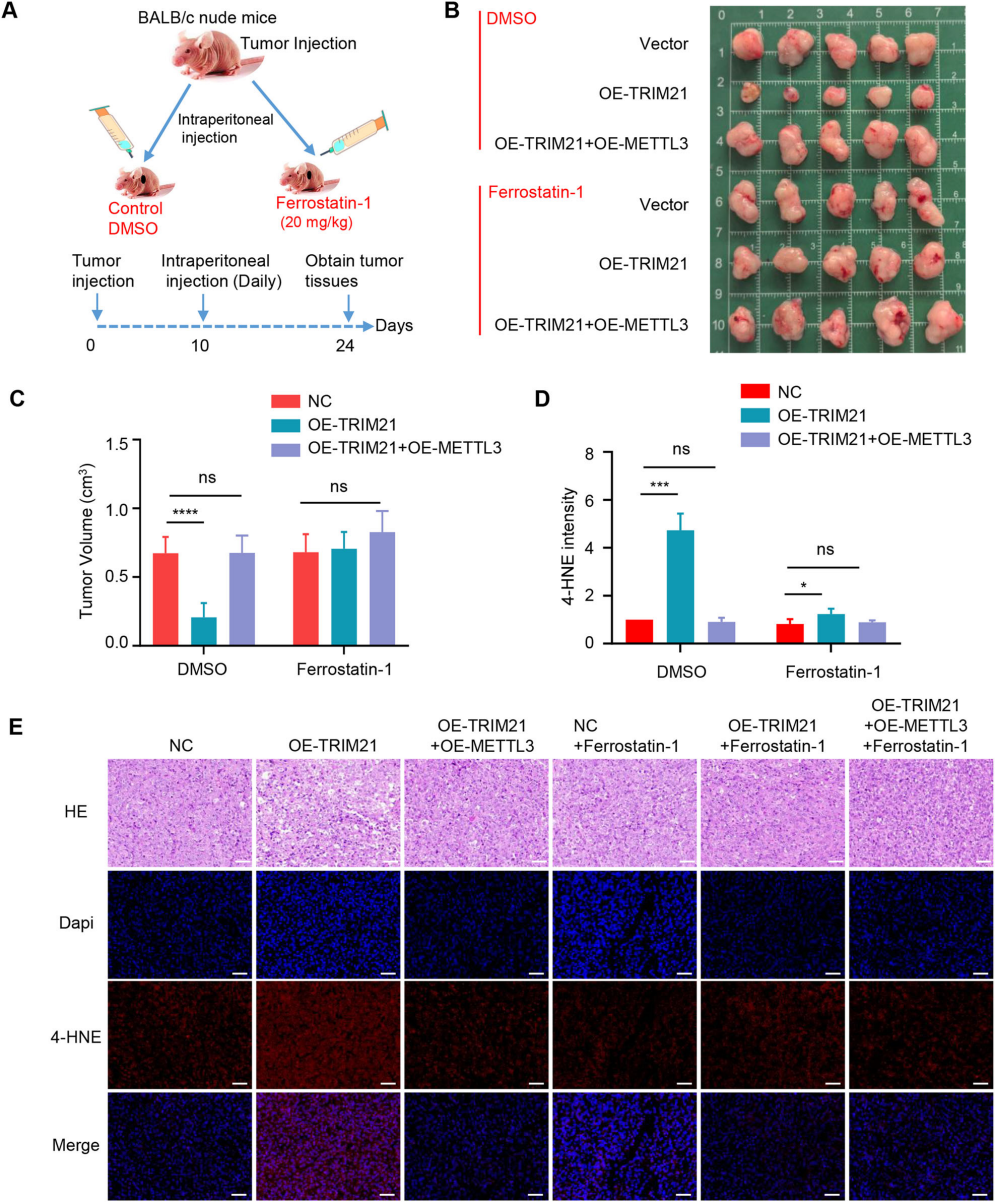
TRIM21 promotes ferroptosis by targeting METTL3 in vivo. **A** Treatment scheme for subcutaneous tumor models. **B** Tumors were excised and photographed. **C** Differences in tumor volume in response to different treatments (*n* = 5). **D** Quantification of immunofluorescence tissue staining with anti-4-HNE antibody. **E** Hematoxylin and eosin (H&E) staining and immunofluorescence staining with anti 4-HNE antibody. Scale bar, 50 µm. (*P < 0.05, **P < 0.01, ***P < 0.001, ****P < 0.0001, ns not significant).

We next investigated the effects of TRIM21 promotes ferroptosis by targeting METTL3 as an intervention for pancreatic cancer progression in vivo (Fig. 6A). TRIM21 overexpression significantly suppressed tumor growth in mouse xenograft model, which was completely rescued by the restored expression of METTL3. This is equivalent to the result of treatment with the ferroptosis inhibitor Ferrostatin-1, which led to substantial rescue of tumor growth suppressed by TRIM21 overexpression, indicating that ferroptosis was indeed involved in TRIM21-regulated tumor growth (Fig. 6B, C). Morphological analysis revealed that TRIM21 overexpression markedly increased the cell death phenotype, which was characterized by cell membrane damage and cytoplasmic vacuolization (Fig. 6E). Staining with anti-4-HNE antibody revealed that METTL3 expression or Ferrostatin-1 also abolished the effects of TRIM21 overexpression on lipid peroxidation (Fig. 6D, E), without influencing cell proliferation indicated through IHC staining with Ki67 (Supplementary Fig. S9E). These results suggest that TRIM21 promotes ferroptosis through the METTL3/SLC7A11 axis in pancreatic cancer and this axis may be a promising target for pancreatic cancer treatment.

### TRIM21-METTL3 axis promotes the therapeutic efficacy of PD-1 antibodies

**Fig. 7:**
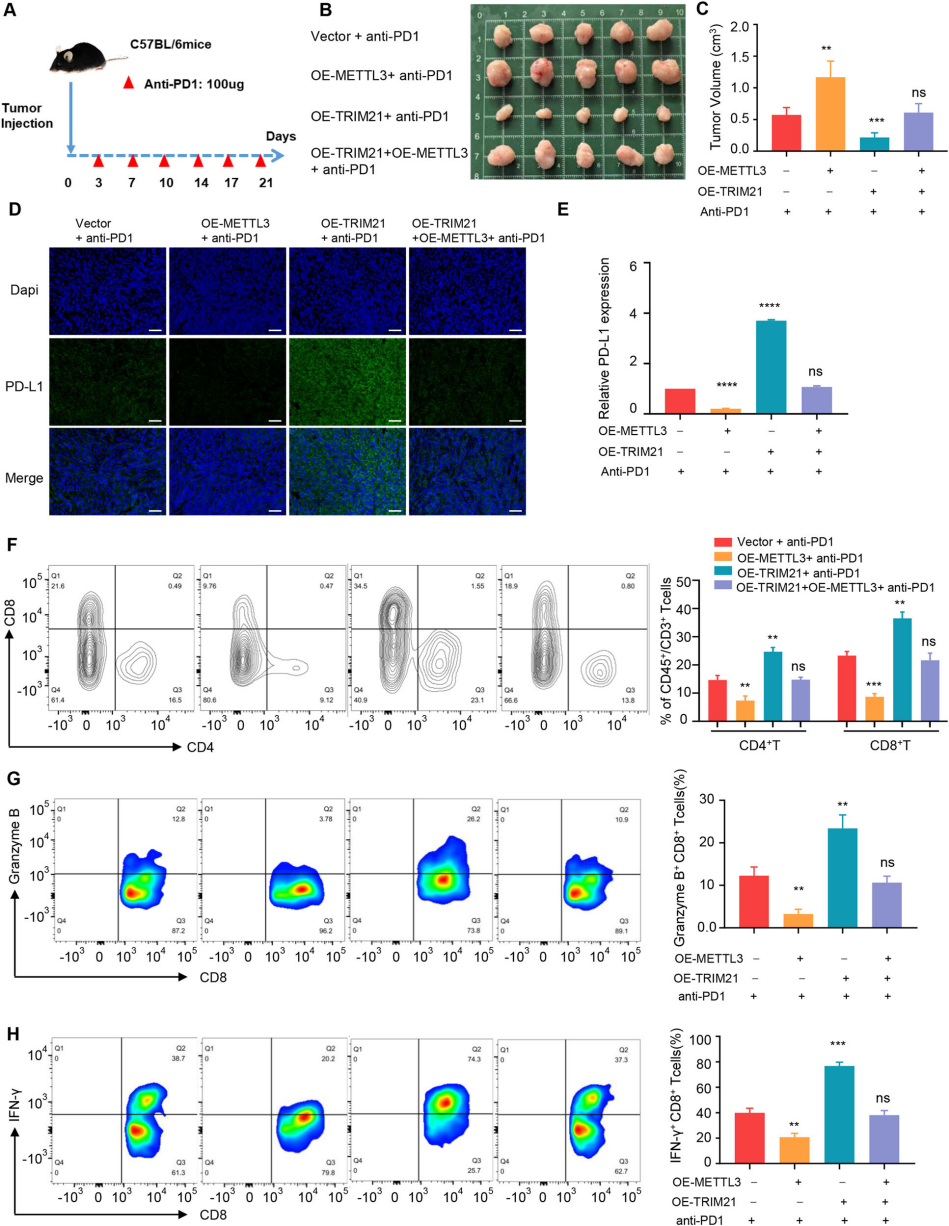
TRIM21-METTL3 axis promotes the therapeutic efficacy of PD-1 antibodies. **A** Schematic showing the treatment schedule to evaluate anti–mPD-1 treatment in different groups. **B** Tumors were dissected and photographed. **C** Differences in tumor volume in response to different treatments (*n* = 5). **D**, **E** IF staining and quantification showing the expression of PD-L1 in different groups. Scale bar, 50 µm. **F** Flow cytometry analysis exhibiting the proportions of infiltrating CD4^+^ and CD8^+^ cells in tumor tissues in response to different groups. **G**, **H** Granzyme B and IFN-γ expression in CD8^+^ T cells from anti PD-1–treated tumors. (*P < 0.05, **P < 0.01, ***P < 0.001, ****P < 0.0001, ns not significant).

Previous findings revealed the close correlation between tumor cell ferroptosis and immune infiltration, which indicate the responsiveness to immunotherapy. In addition to the known ferroptosis effect caused by the activation of TRIM21-METTL3 axis in tumor cells, in this study, we next validated the consequences of TRIM21-METTL3 mediated ferroptosis on the efficacy of anti PD-1 immunotherapy in pancreatic cancer. PANC-02 cells with stably transfected were subcutaneously implanted into C57BL/6 mice, combined with 100 µg anti-PD-1 antibody administered intraperitoneally on indicated days (Fig. 7A). The combination therapy greatly suppressed tumor growth in TRIM21 overexpression tumors, but the therapeutic efficacy of immunotherapy was significantly impaired in METTL3 high expression tumors (Fig. 7B, C). It inspired us to evaluate the potential alteration of PD-L1 expression in tumors of different processing groups. IF staining confirmed highest expression of immune checkpoints PD-L1 on TRIM21 overexpression tumors in comparison to other groups, indicating TRIM21 could promote tumor immunogenicity in pancreatic cancer (Fig. 7D, E). Subsequent FCM revealed that METTL3 overexpression significantly reversed the TRIM21-induced increase in immune infiltration of CD8^+^ T cells (Fig. 7F). Additionally, Granzyme B and IFN-γ are known markers of effector T cells with cytotoxic activity. As shown in Fig. 7G, H, combined treatment with anti PD-1 and TRIM21 overexpression increased the cytotoxic activity of CD8^+^ T cells, while METTL3 overexpression partially reversed TRIM21 effects on immune function. Taken together, these results revealed that TRIM21 increased PD-L1 expression and significantly enhanced antitumor immunity to impede tumor progression via regulating METTL3 in pancreatic cancer.

## Discussion

In recent years, there has been a variety of research on METTL3 involved in the regulation of multiple biological processes [9]. It has been discovered that the METTL3 protein undergoes several post-translational modifications. For example, the SUMOylation of METTL3 was reported to repress its m6A methyltransferase activity without affecting its protein stability, localization, or interaction with METTL14 [14, 15]. Acetylation of METTL3 has been found to influence its translocation between the cytoplasm and nucleus [16]. Moreover, lactylation-driven METTL3-mediated m6A RNA modification has been found to promote the immunosuppression of tumor-infiltrating myeloid cells [17]. Currently, no studies have identified post-translational modifications regulating METTL3 protein stability and the detailed molecular mechanisms underlying METTL3 protein degradation are still not completely understood, which are important for understanding the mechanism of METTL3 functions in tumorigenesis and tumor progression.

**Fig. 8:**
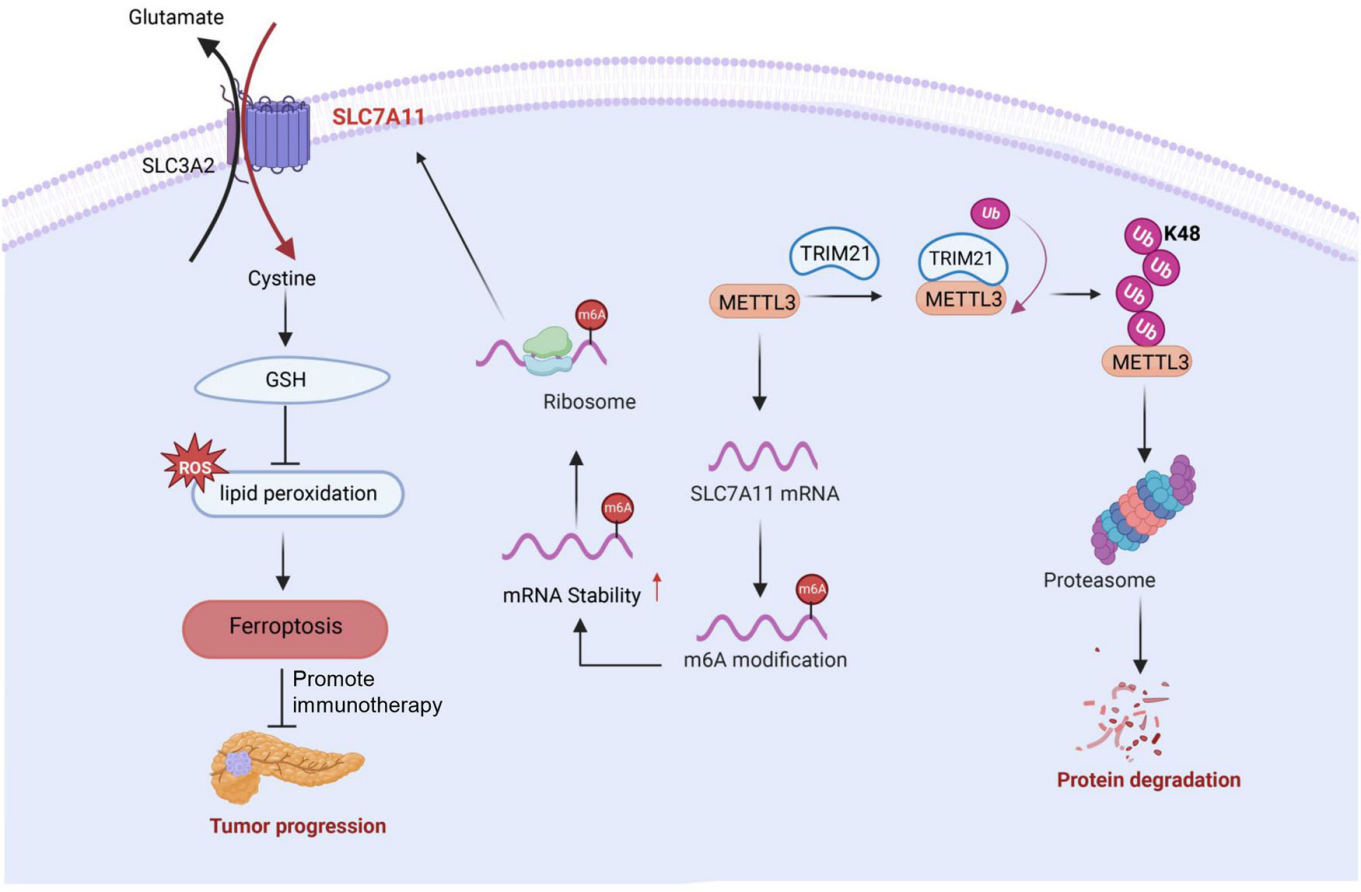
A proposed molecular model. TRIM21-mediated METTL3 degradation promotes PDAC ferroptosis and enhances the efficacy of Anti-PD-1 immunotherapy.

Ferroptosis is inhibited in multiple human cancers and functions as a dynamic tumor suppressor in cancer progression. Sorafenib, sulfasalazine, statins and artemisinin are drugs approved for use in clinical cancer therapy, that can suppress tumor growth by activating ferroptosis [36, 37]. Therefore, ferroptosis has attracted enormous attention and provided new opportunities for cancer therapy. TRIM21 was originally identified as a regulator of innate immune signaling, whereas increasing evidence suggests the versatile functions of TRIM21 in various biological processes, including cell death, cell metabolism, tumorigenesis and redox regulation [38–41]. Interestingly, recent studies identified the potential role of TRIM21 in ferroptosis. Liu et al. observed that TRIM21 regulates ferroptosis by altering the stability of GPX4 protein in colon cancer [42]. Besides, TRIM21 could improve the toxicity of chemotherapeutic agents by regulating ferroptosis responsiveness in cancer. TRIM21 negatively regulates the p62-Keap1-Nrf2 antioxidant pathway and TRIM21 knockout alleviates cardiotoxicity induced by doxorubicin through suppressing ferroptosis [43]. TRIM21-regulated ferroptosis exerts remarkable influence on tumor progression and therapeutic strategies. METTL3, as an oncogene, influences the initiation and progression of various cancers. Accumulating evidence has shown that METTL3 regulates ferroptosis in various cancers by modulating key factors such as SLC7A11, GPX4 and FSP1. In lung adenocarcinoma and hepatoblastoma, METTL3-mediated m6A modification could suppress ferroptosis and promote tumor progression by regulating SLC7A11 mRNA stability and translation [44, 45]. METTL3-mediated m6A methylation of GPX4 prevents ferroptosis in Glioblastoma cells and ultimately promoting tumor progression [46]. METTL3 regulates ferroptosis of non-small cell lung carcinoma through the expression of FSP1 in an m6A manner [47]. In pancreatic cancer, METTL3 plays a crucial role in promoting tumor progression and is positively correlated with poor prognosis. However, there is currently few research confirming that METTL3 or TRIM21 regulates the ferroptosis process in pancreatic cancer. Our study aims to explore the regulatory mechanisms of ferroptosis and reveal potential therapeutic strategies in pancreatic cancer. We identified TRIM21 as the E3 ligase mediating METTL3 degradation was crucial for inducing ferroptosis and suppressing tumorigenesis by disrupting SLC7A11 mRNA stability (Fig. 8). TRIM21 acts as a tumor suppressor by promoting ferroptosis in pancreatic cancer, suggesting that targeting the TRIM21-METTL3-SLC7A11 axis may be a novel effective therapeutic strategy for pancreatic cancer.

Recent studies found that induced ferroptosis could augment antitumor immune responses. Zhang et al. discovered that TSPO inhibition promotes ferroptosis and increases the infiltration and cytotoxicity of CD8^+^ T cells [48]. Besides, TYRO3 suppresses tumor cell ferroptosis and promotes tumor survival during anti–PD-1 therapy [49]. In this study, we investigated the role and underlying mechanism of the TRIM21-METTL3 axis in promoting ferroptosis and cytotoxic activity of CD8^+^ T cells, providing favorable evidence for the connection between ferroptosis and antitumor immunity. Our results indicate that the TRIM21-METTL3 axis may be an important new target for PDAC immunotherapy. However, further studies are required to elucidate the detailed underlying mechanisms.

This study has the potential to provide valuable insights for METTL3 degradation. Most METTL3-related research predominantly focuses on its methyltransferase activity in cancer, however, the precise molecular mechanisms of protein degradation are unclear. We first identified the E3 ligase TRIM21 mediates the K48-linked-polyubiquitination of METTL3 at the K459 site, leading to proteasomal degradation of METTL3, which enriches the PTM regulatory network of METTL3 and revealed the detailed mechanism of METTL3 degradation. Furthermore, The TRIM21-METTL3 axis positively regulates ferroptosis by disrupting SLC7A11 mRNA stability. It also highlights the influence of PTMs in cancer progression. TRIM21-mediated METTL3 ubiquitination as a critical regulator of ferroptosis to prevent tumor progression. Finally, our study revealed the TRIM21-METTL3 axis increases PD-L1 expression and significantly enhanced antitumor immunity. Immunotherapy has limited effects in the clinical treatment of pancreatic cancer [4]. Our findings provide new insights into a connection between ferroptosis induction and immunotherapy in pancreatic cancer, which establish a combination therapy for the cancer clinical treatment.

However, there are still some limitations in our work. First, the upstream mechanism that regulates TRIM21 is less well defined. Besides, the detailed underlying mechanisms of the TRIM21-METTL3 axis promotes the cytotoxic activity of CD8^+^ T cells remains unclear. Further studies are needed to explore the more complete specific mechanism in the future.

## Supplementary information

Supplementary Tables

Supplementary Figures

Original western blots

## Data Availability

All data supporting the conclusions in this article are present in the paper and/or the Supplementary Materials. The public datasets used in this study were obtained from Gene Expression Omnibus (GEO) GSE71989 and GSE15471 and GSE183795. Additional data related to this article may be requested from the corresponding author.
