# Supplementary material for: TRIM21-mediated METTL3 degradation promotes PDAC ferroptosis and enhances the efficacy of Anti-PD-1 immunotherapy
Source: Cell Death Dis. 2025 Apr 3;16(1):240. doi: 10.1038/s41419-025-07550-y (PMC11965403; doi:10.1038/s41419-025-07550-y)
Supplement: Supplementary file 1 — Supplementary Tables [file 41419_2025_7550_MOESM1_ESM.docx]

**Supplementary Tables**

**Table S1. Experimental related reagents.**

| **Reagents** | **Sourse** | **Manufacturer** | **Identifier** |
| --- | --- | --- | --- |
| MG132 | Selleck | USA (Houston) | S2619 |
| Cycloheximide (CHX) | GlpBio | USA (Montclair) | GC17198 |
| Geneticin, G-418 Sulfate | GlpBio | USA (Montclair) | GC17427 |
| Cell Counting Kit-8 (CCK-8) | GlpBio | USA (Montclair) | GK10001 |
| Puromycin | Yeasen | China (Shanghai) | 60209ES10 |
| Polybrene | Yeasen | China (Shanghai) | 40804ES76 |
| Chloroquine | MedChemExpress | USA (New Jersey) | HY-17589A |
| Actinomycin D | MedChemExpress | USA (New Jersey) | HY-17559 |
| Necrostatin 1 | Selleck | USA (Houston) | S8037 |
| Erastin | Selleck | USA (Houston) | S7242 |
| Ferrostatin-1 | Selleck | USA (Houston) | S7243 |
| Protease Inhibitor Cocktail | Selleck | USA (Houston) | B14001 |
| IPTG | Sangon | China (Shanghai) | A600168 |
| Lipid Peroxidation Probe BDP 581/591 C11 | DOJINDO | Japan (Kyushu Island) | L267 |
| Lipid Peroxidation MDA Assay Kit | Beyotime | China (Shanghai) | S0131S |
| GSH and GSSG Assay Kit | Beyotime | China (Shanghai) | S0053 |
| NP-40 Lysis Buffer | Beyotime | China (Shanghai) | P0013F |
| RIPA Lysis Buffer | Beyotime | China (Shanghai) | P0013B |
| Protein A/G PLUS-Ararose | Santa Cruz | USA (California) | SC-2003 |
| Glutathione-Agarose | Santa Cruz | USA (California) | SC-2009 |
| PI | Thermofisher | USA (Massachusetts) | P3566 |
| Lipofectamine™ 3000 | Invitrogen | USA (California) | L3000075 |
| Protein G for Immunoprecipitation | Invitrogen | USA (California) | 10003D |
| Ni-NTA Beads | Smart-lifesciences | China (Changzhou) | SA004GC01 |
| Cell-Light EdU Kit | RiboBio | China (Guangzhou) | C10310-1 |

**Table S2. Experimental related shRNAs.**

| **sh-RNA** | **sense（5'-3'）** | **Sourse** |
| --- | --- | --- |
| shMETTL3-1 | GCAAGAATTCTGTGACTAT | GenePharma |
| shMETTL3-2 | CCUGCAAGUAUGUUCACUA | GenePharma |
| shTRIM21-1 | UCAUUGUCAAGCGUGCUGC | GenePharma |
| shTRIM21-2 | UGGCAUGGAGGCACCUGAAGGUGG | GenePharma |

**Table S3. Primers used for quantitative PCR.**

| **q-PCR Primers** | **Sequence(5’-3’)** | **Sourse** |
| --- | --- | --- |
| hGAPDH-F | CAGGAGGCATTGCTGATGAT | Tsingke Biotechnology |
| hGAPDH-R | GAAGGCTGGGGCTCATTT | Tsingke Biotechnology |
| hSLC7A11-F | ATGCAGTGGCAGTGACCTTT | Tsingke Biotechnology |
| hSLC7A11-R | GGCAACAAAGATCGGAACTG | Tsingke Biotechnology |
| hTRIM21-F | TCAGCAGCACGCTTGACAAT | Tsingke Biotechnology |
| hTRIM21-R | GGCCACACTCGATGCTCAC | Tsingke Biotechnology |
| hMETTL3-F | TTGTCTCCAACCTTCCGTAGT | Tsingke Biotechnology |
| hMETTL3-R | CCAGATCAGAGAGGTGGTGTAG | Tsingke Biotechnology |
| hGPX4-F | GCCTTCCCGTGTAACCAGT | Tsingke Biotechnology |
| hGPX4-R | GCGAACTCTTTGATCTCTTCGT | Tsingke Biotechnology |

**Table S4. Experimental related antibodies**

| **Antibodies** | **Sourse** | **Identifier** |
| --- | --- | --- |
| Ubiquitin Mouse mAb | Cell Signaling Technology | #3936 |
| m6A antibody | Synaptic Systems | 202 003 |
| METTL3 Polyclonal antibody | Proteintech Group | 15073-1-AP |
| METTL3 Monoclonal antibody | Proteintech Group | 67733-1-Ig |
| TRIM21 Polyclonal antibody | Proteintech Group | 12108-1-AP |
| HRP-conjugated Affinipure Goat Anti-Mouse IgG(H+L) | Proteintech Group | SA00001-1 |
| HRP-conjugated Affinipure Goat Anti-Rabbit IgG(H+L) | Proteintech Group | SA00001-2 |
| Alexa Fluor 488-labeled Goat Anti-Rabbit IgG(H+L) | beyotime | A0423 |
| Cy3-labeled Goat Anti-Mouse IgG (H+L) | beyotime | A0521 |
| His-Tag Antibody | Abmart | M20001 |
| DYKDDDDK-Tag mAb (Anti-FLAG) | Abmart | M20008 |
| HA-Tag mAb | Abmart | M20003 |
| GPX4 antibody | Abmart | T56959 |
| SLC7A11 antibody | Abmart | T57046 |
| Goat anti-Mouse IgG HRP  (avoid heavy chain antibody) | Abmart | M21004 |
| Mouse anti-Rabbit IgG HRP  (avoid heavy chain antibody) | Abmart | M21006 |
| Anti- anti-mouse PD-1 antibody | BioXCell | BE0273 |
| Anti-mouse CD16/32 | BioLegend | 101302 |
| FITC-anti-mouse CD3 | BioLegend | 100203 |
| APC-anti-mouse CD8 | BioLegend | 100711 |
| APC/Cyanine anti-mouse CD45 | BioLegend | 103115 |
| Brilliant Violet 605^TM^ anti-mouse CD4 | BioLegend | 100547 |
| Brilliant Violet 785 ^TM^ anti-mouse IFN-γ | BioLegend | 505837 |
| PE/Cyanine7 anti-mouse GranzymeB | BioLegend | 372214 |
| Zombie Aqua^TM^ Fixable Viability Kit | BioLegend | 423101 |

**Table S5. Baseline characteristics of the tissue.**

| **characteristics** | **Total (n=90)**  **n%** | **Low (n=44)**  **n%** | **High (n=44)**  **n%** |
| --- | --- | --- | --- |
| **Age（years）** |  |  |  |
| ≤ 60 | 26 | 13 | 12 |
| ＞60 | 64 | 31 | 32 |
| **Gender** |  |  |  |
| Female | 41 | 23 | 18 |
| Male | 49 | 21 | 26 |
| **Histological grade** |  |  |  |
| G1 | 20 | 18 | 2 |
| G2 | 54 | 24 | 28 |
| G3 | 15 | 2 | 13 |
| **Clinical stage** |  |  |  |
| Stage I | 39 | 21 | 17 |
| Stage II | 46 | 23 | 22 |
| Stage III | 0 | 0 | 0 |
| Stage IV | 5 | 0 | 5 |
| **Survival** |  |  |  |
| ＞1-year | 64 | 38 | 24 |
| ＞3-year | 38 | 28 | 8 |
| ＞5-year | 15 | 11 | 3 |
| **OS event** |  |  |  |
| Alive | 29 | 22 | 6 |
| Dead | 61 | 22 | 38 |

The pathological stage of one patient was not detailed. Additionally, the protein expression of METTL3 could not be evaluated due to tissue damage in the pathological sections of two patients.
