# Supplementary material for: TRIM21-mediated METTL3 degradation promotes PDAC ferroptosis and enhances the efficacy of Anti-PD-1 immunotherapy
Source: Cell Death Dis. 2025 Apr 3;16(1):240. doi: 10.1038/s41419-025-07550-y (PMC11965403; doi:10.1038/s41419-025-07550-y)
Supplement: Supplementary file 2 — Supplementary Figures [file 41419_2025_7550_MOESM2_ESM.docx]

**Supplementary Figures**


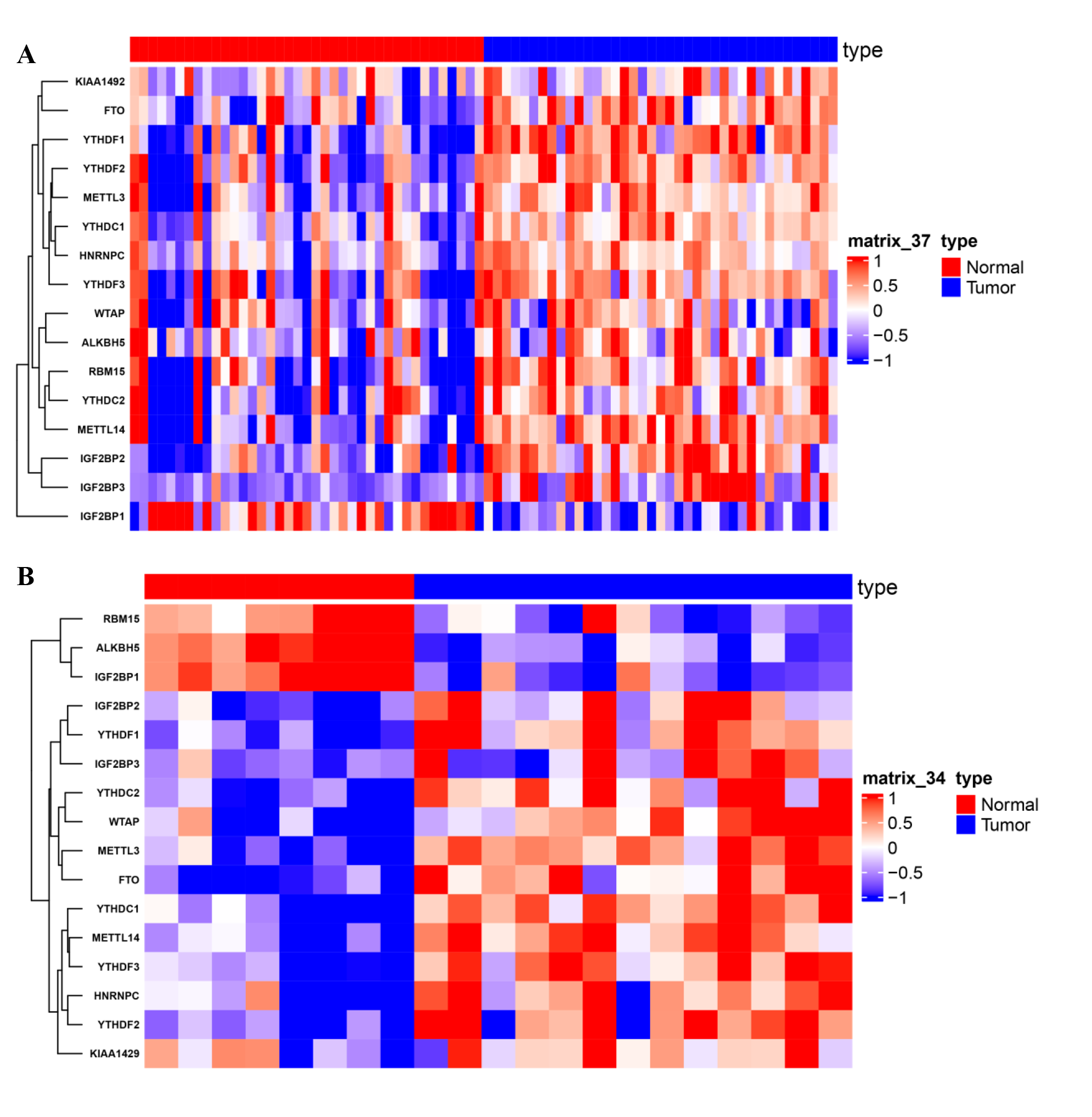
 **Supplementary Figure S1.** **m6A regulators expression in pancreatic cancer.**

**A.B.** The heatmap shows the expression levels of m6A RNA methylation regulators in pancreatic cancer tissues compared to normal tissues in the GEO databases GSE71989 and GSE15471.


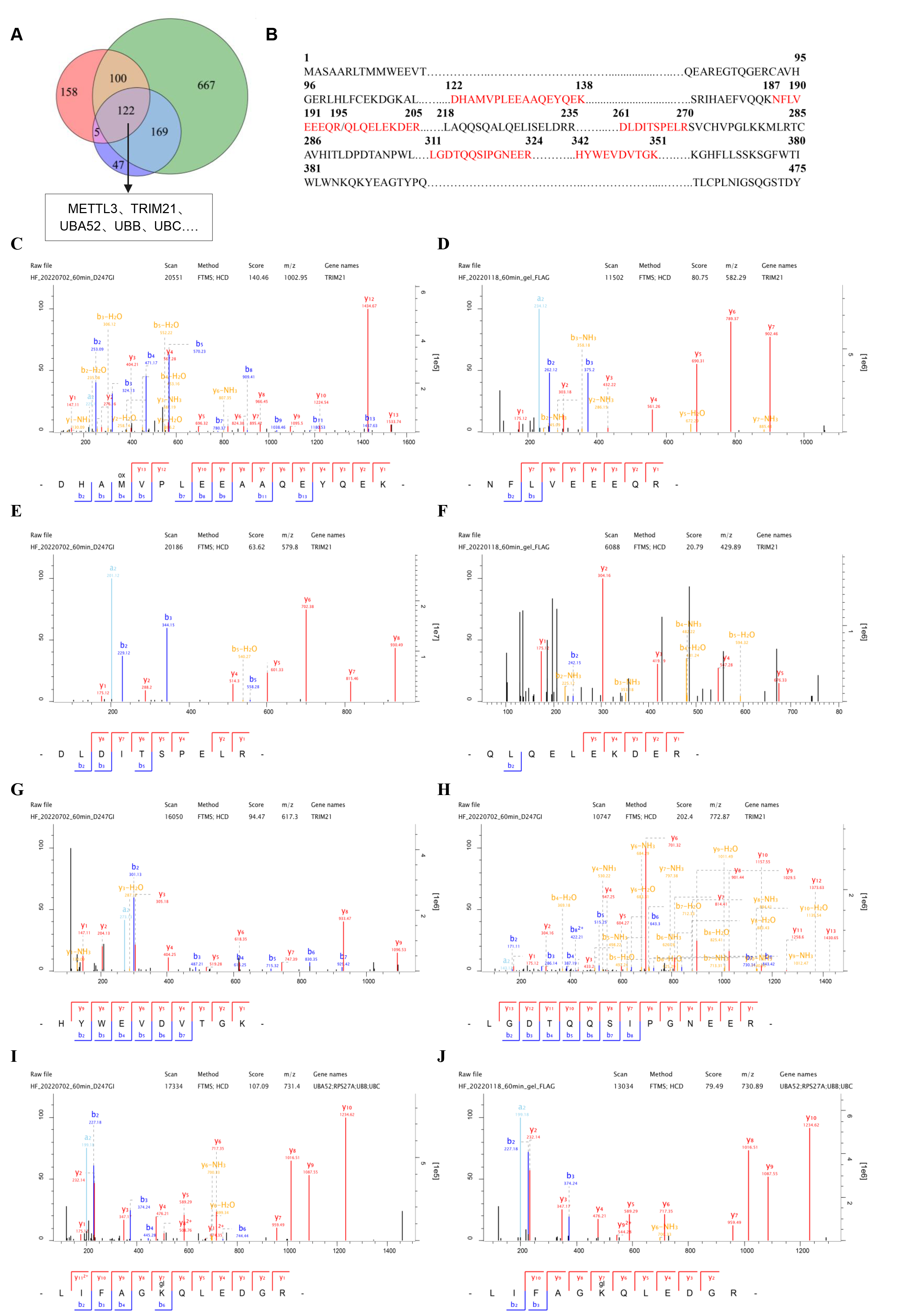


**Supplementary Figure S2.** **LC-MS/MS analysis revealed METTL3-associated proteins**

**A.** Venn diagram shows METTL3 interacting proteins obtained from three independent LC/MS analyses. **B.** Displayed all positional sequence information for the unique peptides of TRIM21 protein obtained by mass spectrometry. **C-H.** LC/MS spectrometry analysis identified six unique peptide sequences of TRIM21 protein. **I.J.** Ubiquitin peptides were detected on LC/MS spectrometry analysis. The ubiquitin protein has a polyubiquitinated modification at K48.


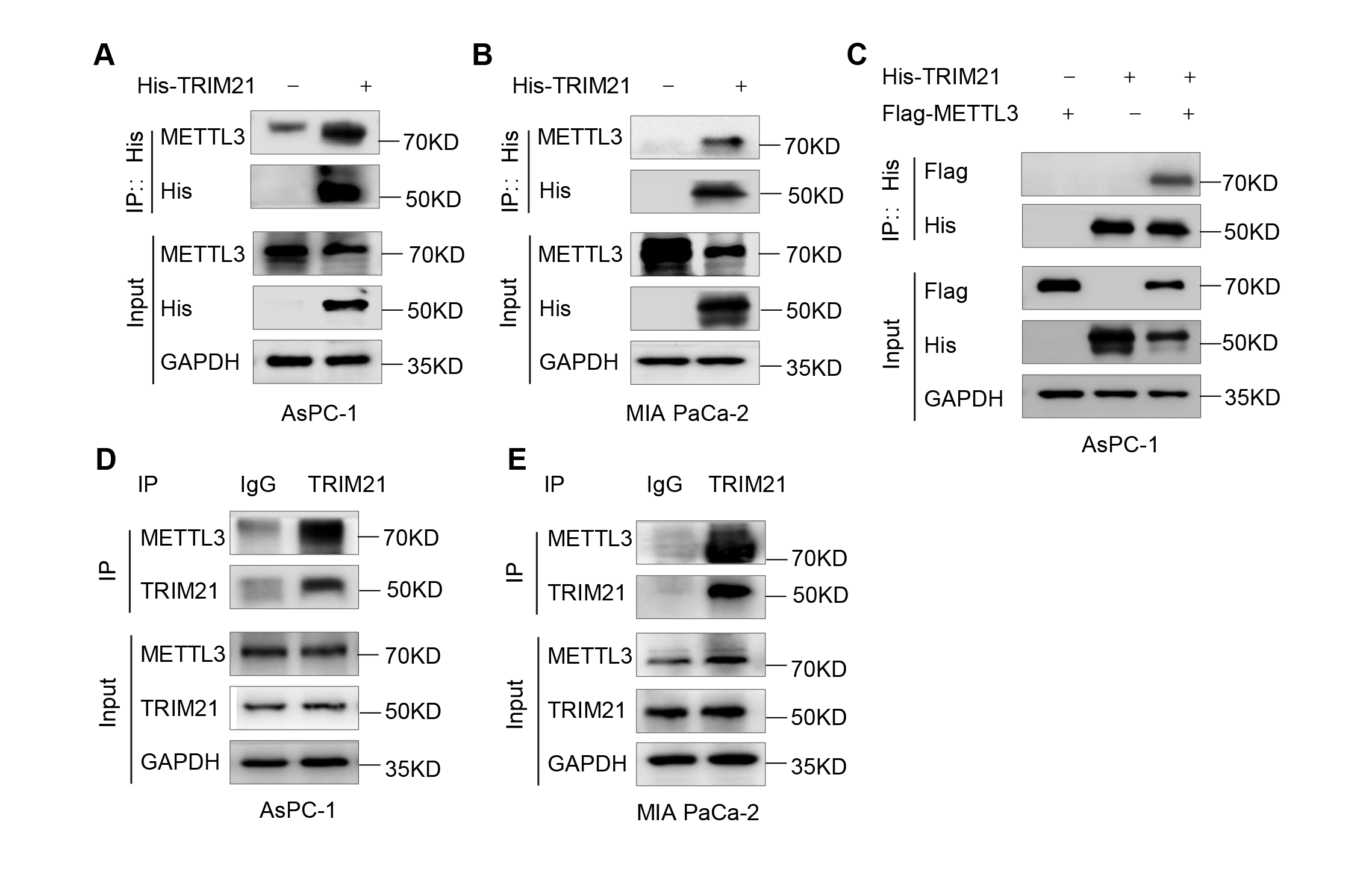


**Supplementary Figure S3. TRIM21 directly interacts with METTL3.**

**A.B.** The interaction between His-tagged TRIM21 and endogenous METTL3 was confirmed by co-IP assay. **C.** co-IP analysis to detect the exogenous protein interaction of Flag-METTL3 and His-TRIM21 in AsPC-1 cells. **D.E.** Immunoprecipitation with anti-TRIM21 antibody was used to verify the interaction between endogenous METTL3 and TRIM21 in pancreatic cancer cells.


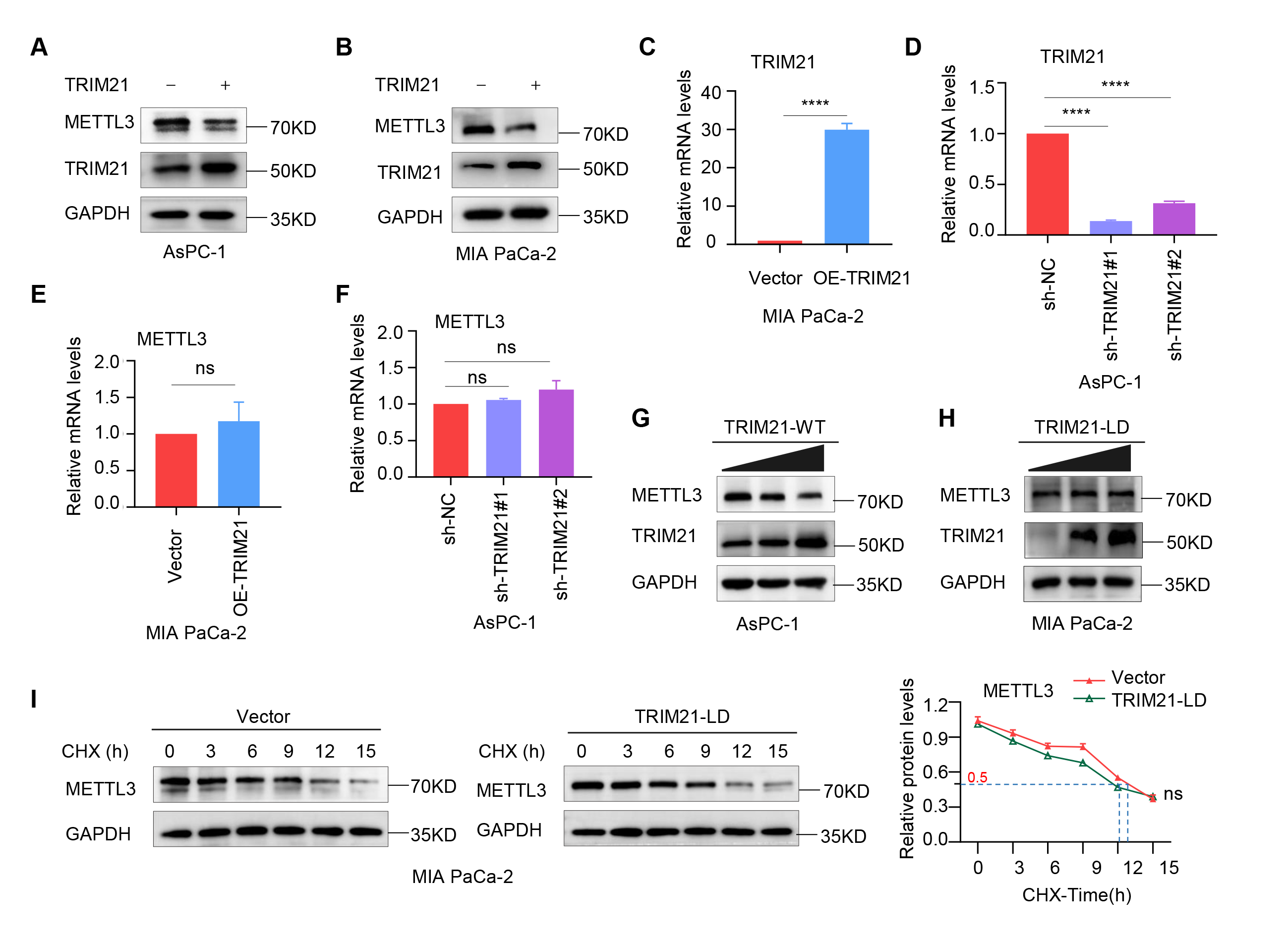


**Supplementary Figure S4. TRIM21 promotes the proteasomal degradation of METTL3**

**A**.**B**. The expression of METTL3 was detected by western blot in pancreatic cancer cells with TRIM21 overexpression or vector plasmids. **C**.**D**. RT-qPCR detected the transfection efficiency of TRIM21 overexpression plasmid and TRIM21-shRNAs. **E**.**F**. RT-qPCR detected the mRNA expression levels of METTL3 in pancreatic cancer cells with altered TRIM21 expression. **G**.**H**. TRIM21-WT or TRIM21-LD mutant plasmids were transfected into AsPC-1 or MIA PaCa-2 cells in a dose-dependent manner, followed by the western blot analysis of METTL3 protein expression. **I**. Western blot analyzed METTL3 degradation in TRIM21-LD mutant or vector transfected cells. (*P <0.05, **P <0.01, ***P <0.001, ****P <0.0001, ns, not significant).


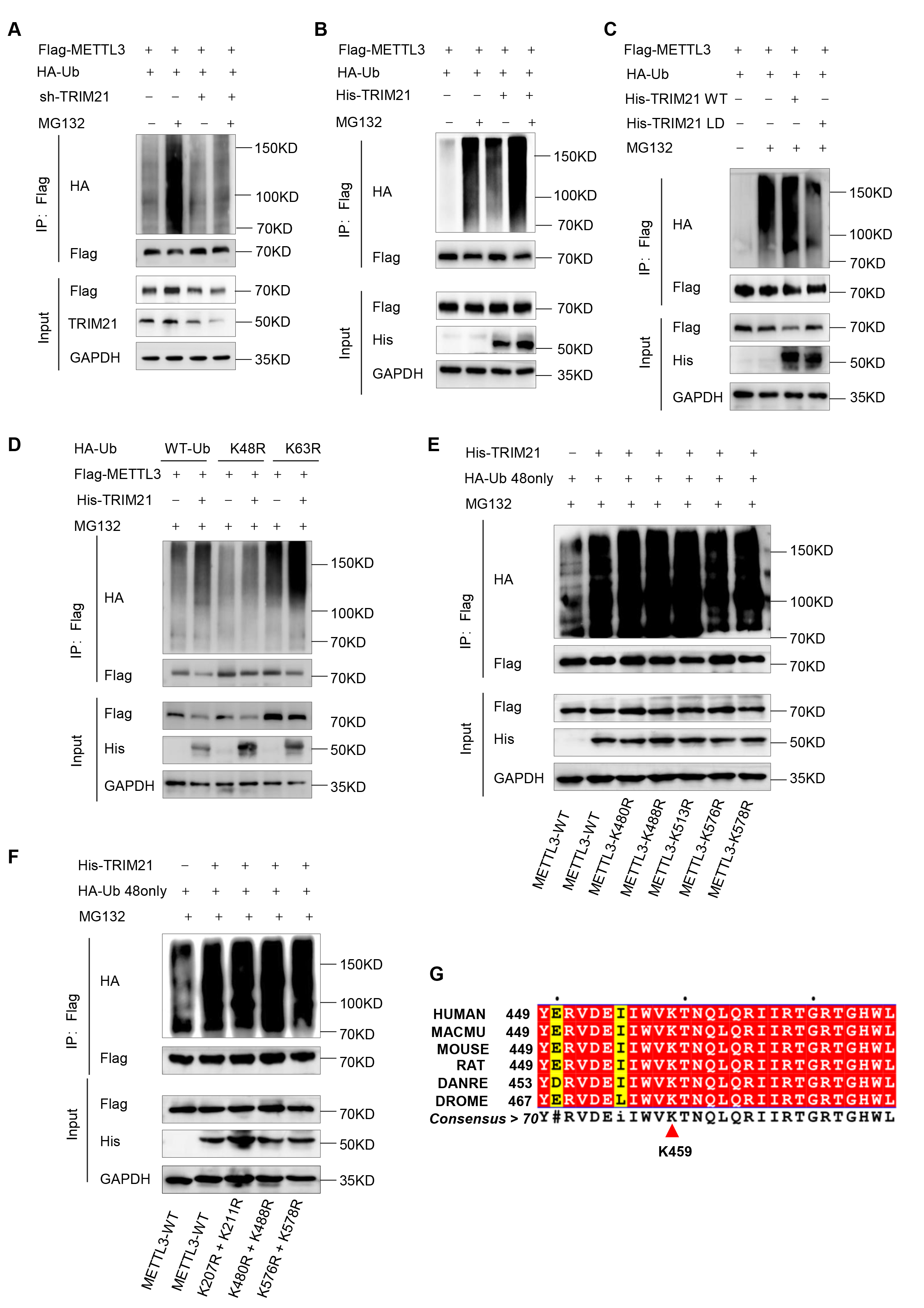


**Supplementary Figure S5. TRIM21 mediates the polyubiquitination of METTL3 at the K459 site**

**A**. IP-IB of ubiquitinated METTL3 using 293T cells transfected with Flag-METTL3, HA-ubiquitin and/or sh-TRIM21 and treated with MG132. **B.** IP-IB of ubiquitinated METTL3 using 293T cells transfected with Flag-METTL3, HA-ubiquitin and/or His-TRIM21 and treated with MG132. **C.** IP-IB of ubiquitinated METTL3 using 293T cells transfected with Flag-METTL3, HA-ubiquitin and/or His-TRIM21 WT or His-TRIM21-LD and treated with MG132. **D.** IP-IB of ubiquitinated METTL3 using 293T cells transfected with Flag-METTL3, HA-Ub (WT, K48R, and K63R) and/or His-TRIM21 and treated with MG132 (10 μM). **E.** Flag-METTL3 and arginine substitution mutants (K480R, K488R, K513R, K576R, K578R) were expressed in 293T cells with His-TRIM21 and HA-Ub K48 only plasmids. Lysates were prepared for IP and western blotting with indicated antibodies. **F.** Immunoprecipitation and western blotting demonstrated the impact of double-mutants on TRIM21-mediated METTL3 ubiquitination. **G**. Sequence analysis of METTL3 protein among various species. The conserved site K459 of METTL3 was marked by arrowhead.


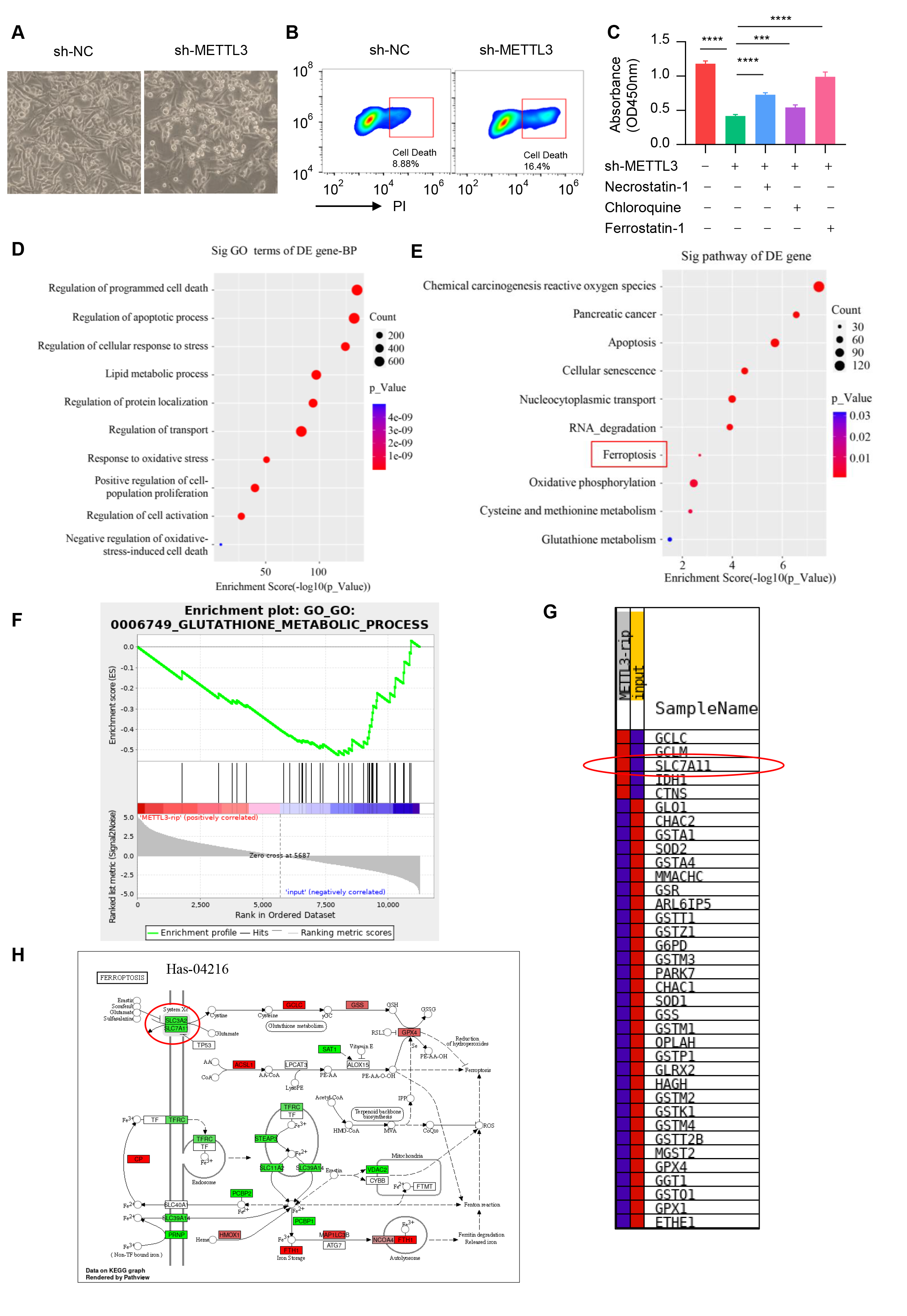


**Supplementary Figure S6. SLC7A11 is a target of METTL3 regulating cell ferroptosis**

**A**. Knocking down METTL3 expression promoted cell death was observed under 10X optical microscope. **B** Flow cytometry analysis was used to determine cell death rates in METTL3-knockdown cells. **C**. CCK8 assay was used to detect the viability in METTL3-knockdown cells with various cell death inhibitors. **D.E**. The GO analysis and KEGG pathway enrichment of METTL3-RIP sequencing. **F**. Gene set enrichment analysis (GSEA) of sequencing data showing that METTL3-RIP-RNAs were involved in the regulation of Glutathione metabolic process. **G**. Detailed target mRNAs from METTL3-RIP sequencing in GSEA of Glutathione metabolic process. **H.** The mechanism of SLC7A11 in the ferroptosis pathway map with KEGG analysis. (*P <0.05, **P <0.01, ***P <0.001, ****P <0.0001).


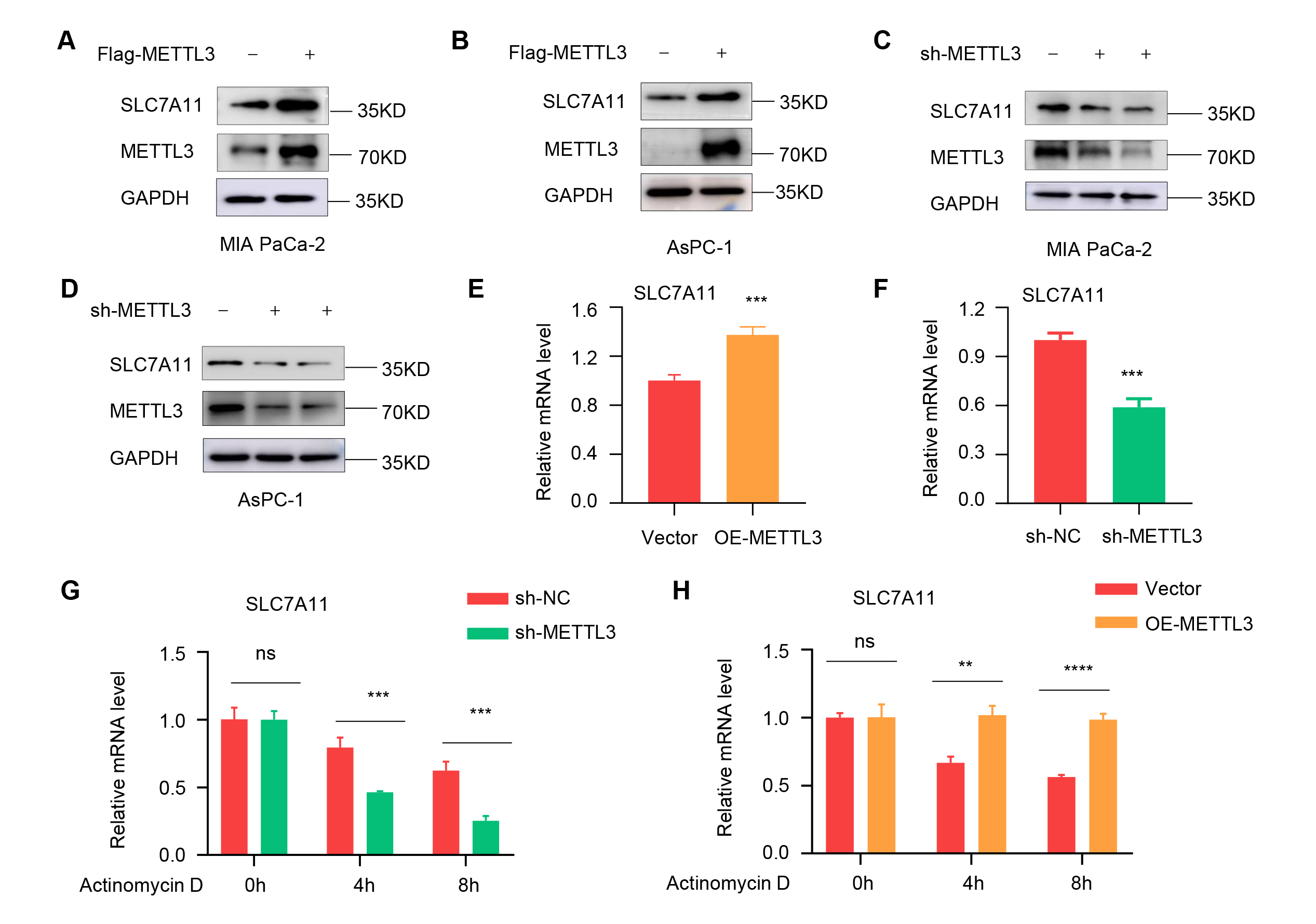


**Supplementary Figure S7. METTL3 regulates SLC7A11 expression by increasing mRNA stability**

**A. B.** Western blot verified the protein levels of SLC7A11 were positively correlated with METTL3 expression in pancreatic cancer cells. **C. D.** Western blot analyzed the effect of METT3 knockdownon on SLC7A11 protein levels. **E.F.** RT-qPCR validated SLC7A11 RNA expression followed METTTL3 alteration in pancreatic cancer cells. **G.H.** RT-qPCR measured the effect of METTL3 expression on SLC7A11 mRNA stability in MIA PaCa-2 cells with actinomycin D (5 μg/mL) treatment. (*P <0.05, **P <0.01, ***P <0.001, ****P <0.0001, ns, not significant).

**
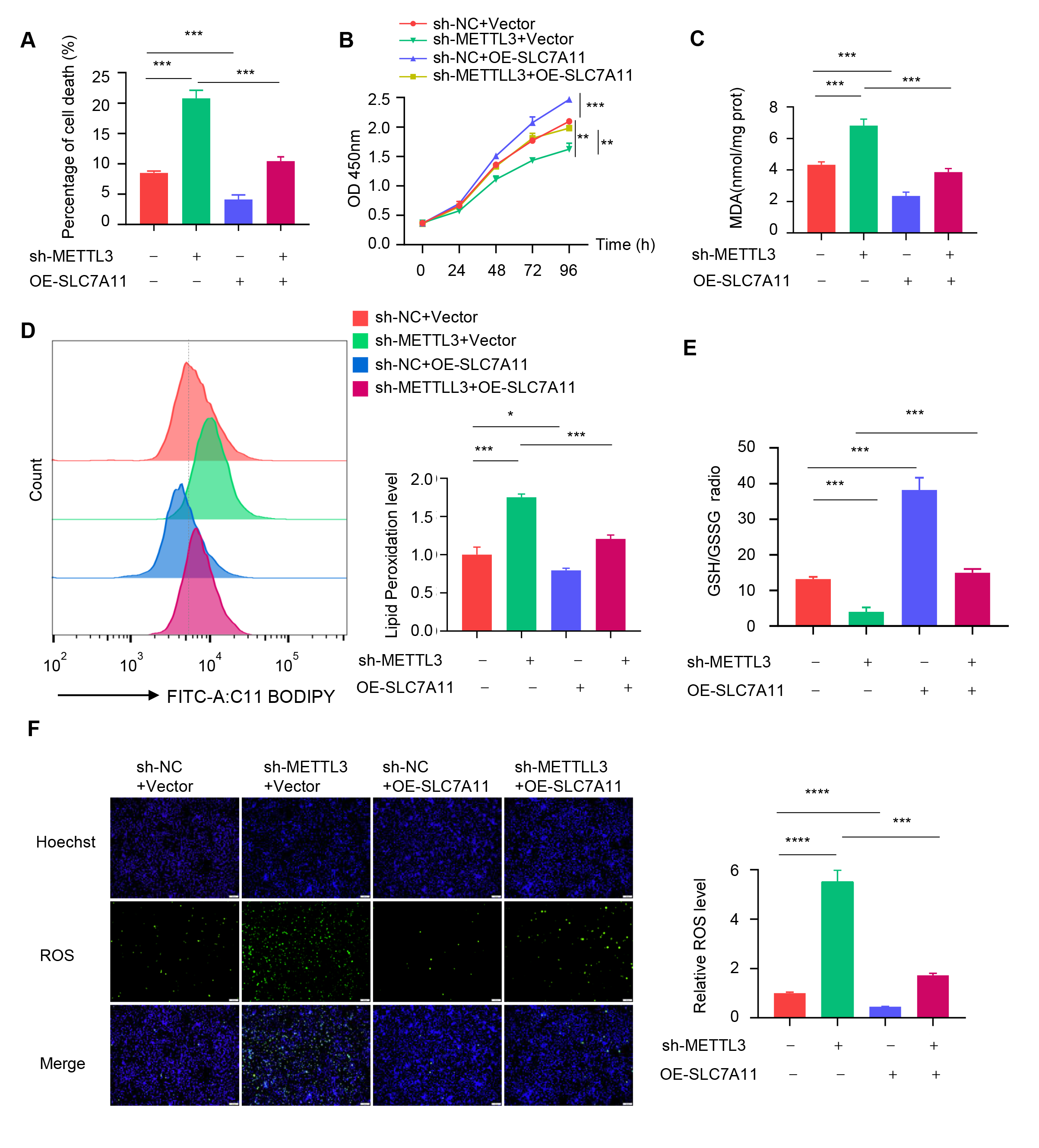
**

**Supplementary Figure S8. METTL3 inhibits cell ferroptosis through SLC7A11**

**A**. Flow cytometry analysis was used to determine cell death rates in MIA Paca-2 cells with METTL3 knockdown cells or/and SLC7A11 overexpression. **B**. CCK8 assay was used to detect cell viability. **C**. The level of intracellular MDA in METTL3 knockdown cells after SLC7A11 overexpression. **D**. lipid peroxidation level was measured by C11-BODIPY581/591 staining with flow cytometry analysis. **E.** The glutathione to oxidized glutathione ratio (GSH/GSSG Ratio) was measured in different groups. **F.** Fluorescence of DFCH-DA probe was measured using fluorescent microscope to detect intracellular ROS levels. Scale bar, 50 μm. (*P <0.05, **P <0.01, ***P <0.001, ****P <0.0001, ns, not significant).


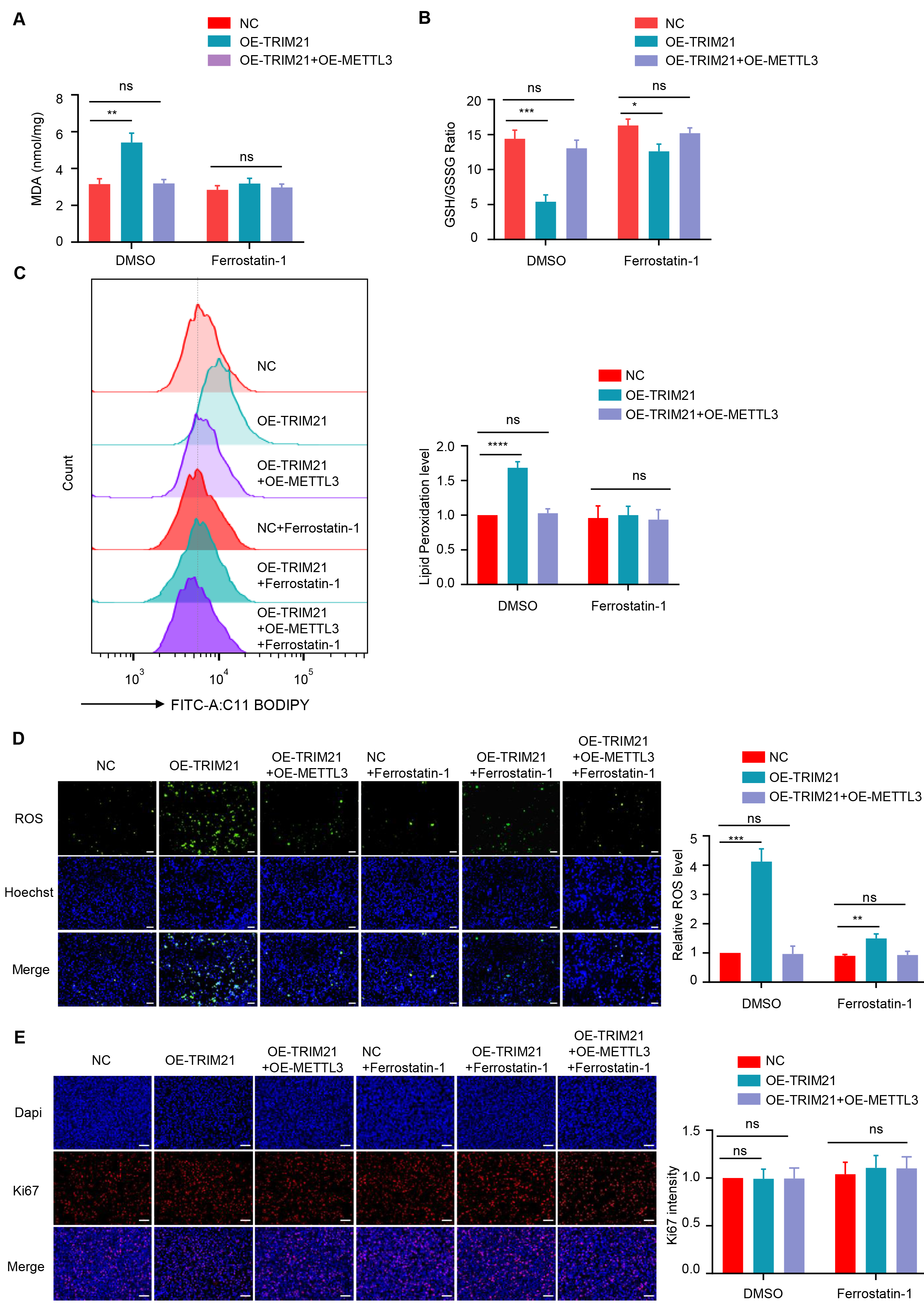


**Supplementary Figure S9. TRIM21 promotes ferroptosis via regulating METTL3-SLC7A11 axis**

**A**. The level of intracellular MDA induced by TRIM21 overexpression was effectively rescued by METTL3 overexpression or treatment with ferroptosis inhibitor ferrostatin-1 (1 μM). **B**. Measurement of the GSH/GSSG ratio in AsPC-1 cells under different treatments. **C**. Lipid peroxidation levels were measured by C11-BODIPY581/591 staining with flow cytometry analysis. **D.** Fluorescence of DFCH-DA probe was measured using fluorescent microscope to detect intracellular ROS levels in subgroups. Scale bar, 50 μm. **E.** Immunofluorescence staining and quantification with anti Ki67. Scale bar, 50 µm. (*P <0.05, **P <0.01, ***P <0.001, ****P <0.0001, ns, not significant).
