# Supplementary material for: TRIM21-mediated METTL3 degradation promotes PDAC ferroptosis and enhances the efficacy of Anti-PD-1 immunotherapy
Source: Cell Death Dis. 2025 Apr 3;16(1):240. doi: 10.1038/s41419-025-07550-y (PMC11965403; doi:10.1038/s41419-025-07550-y)

1.Supplementary materials

Full scans of uncropped blots presented in Figures of the paper
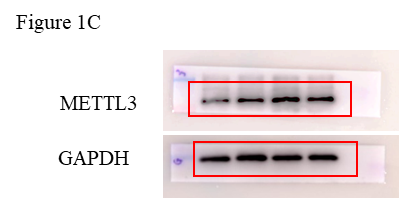

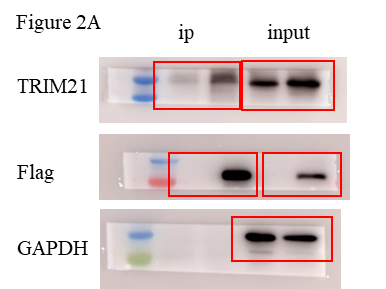

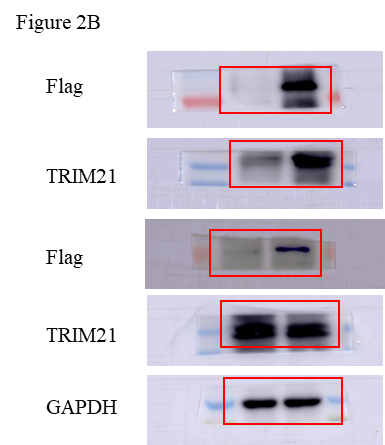

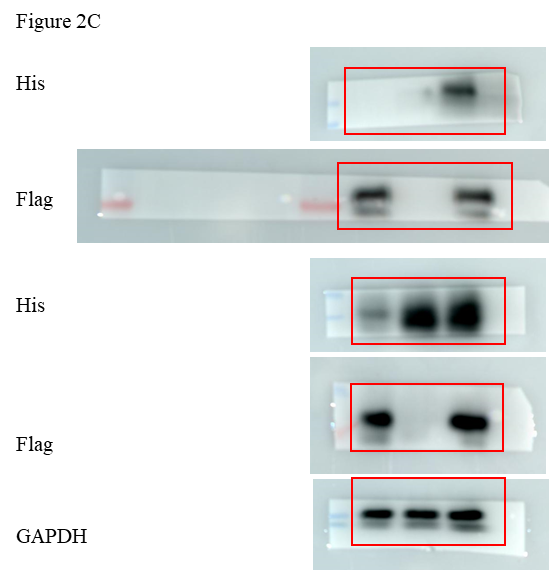

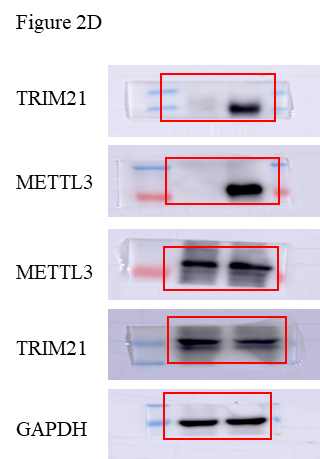

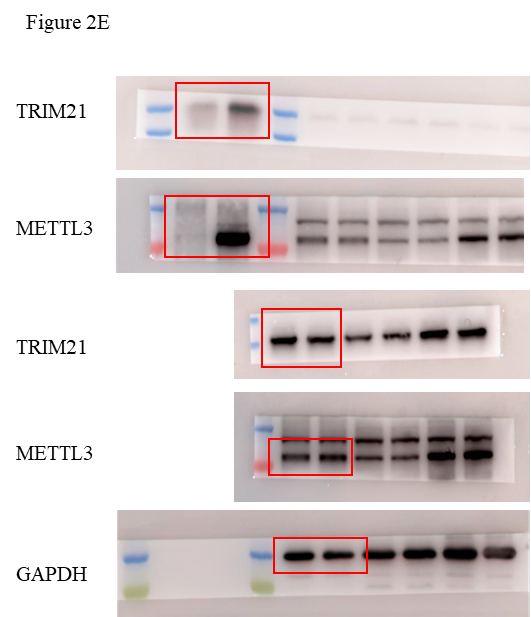


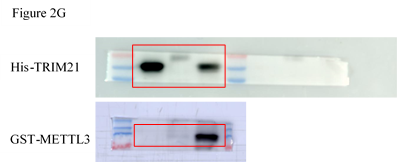


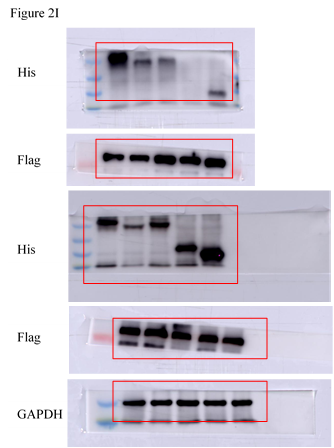


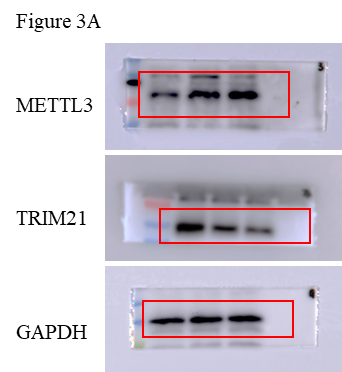

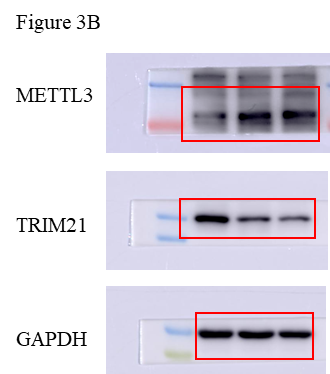


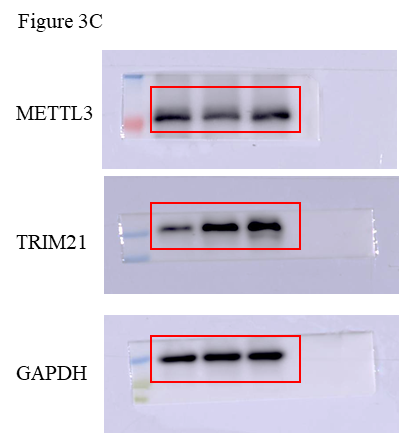

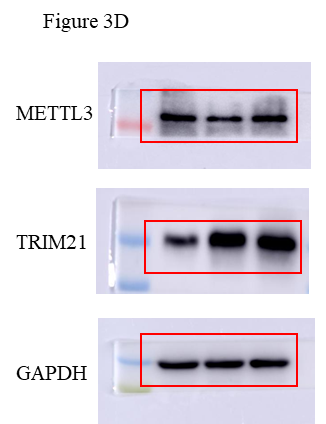


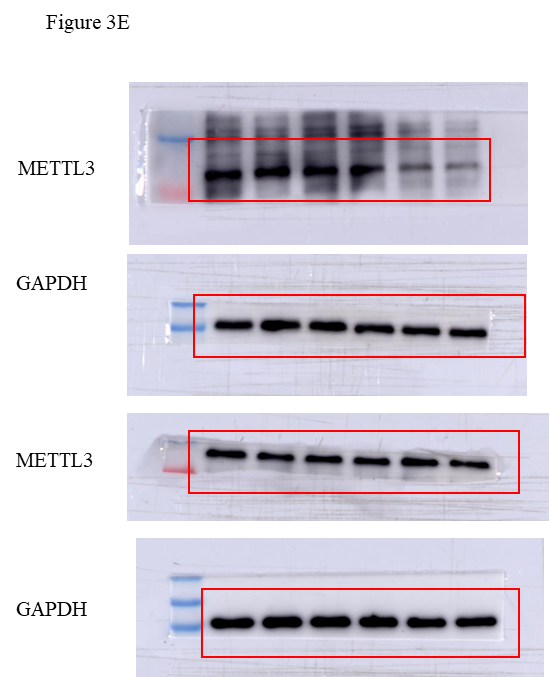


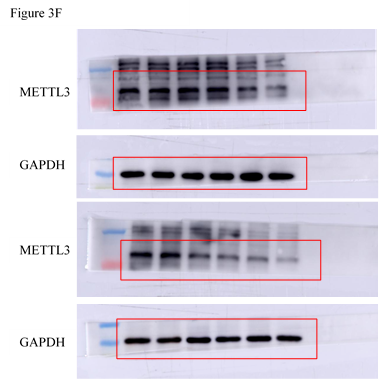

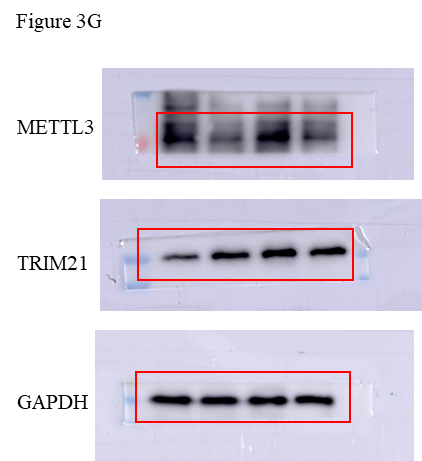

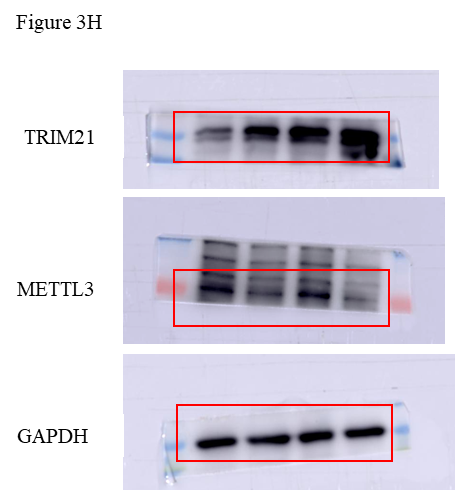


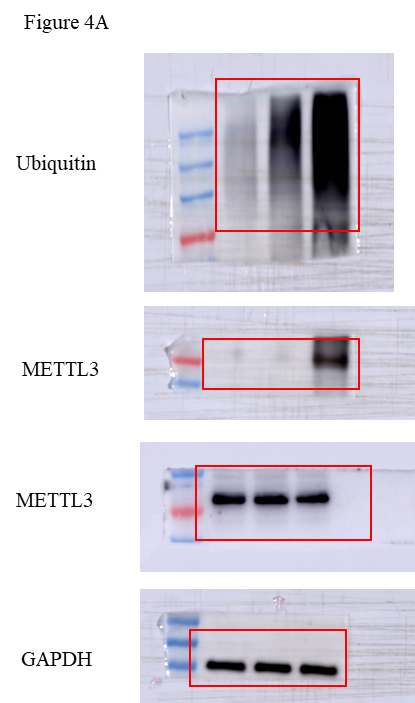

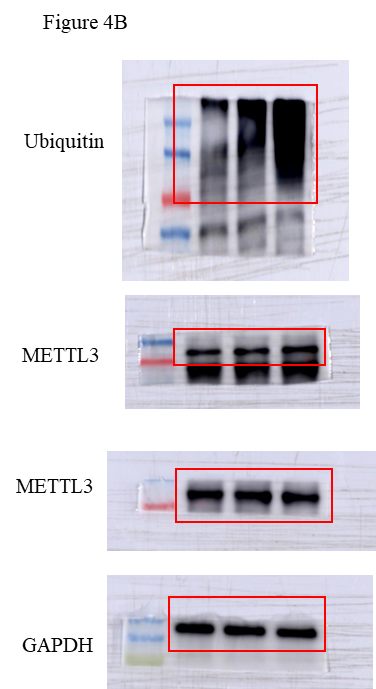


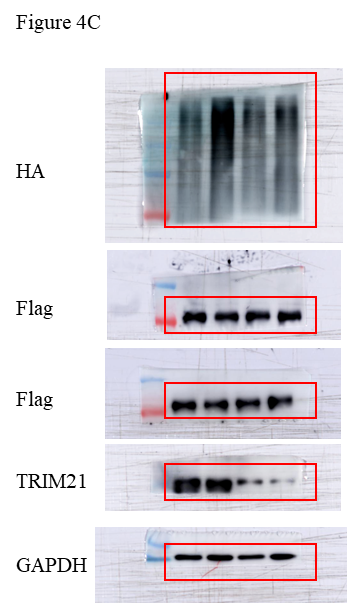

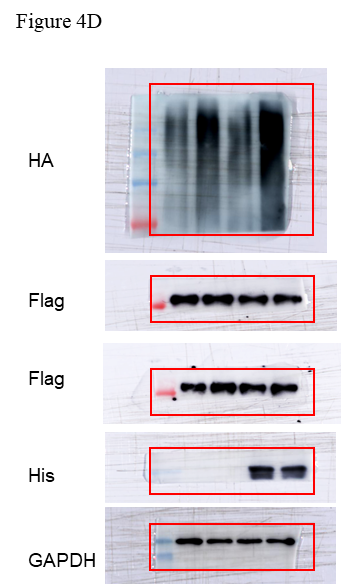

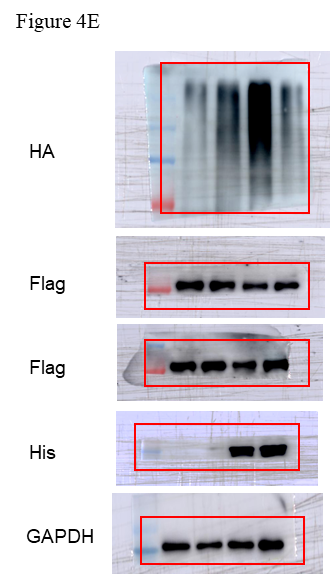

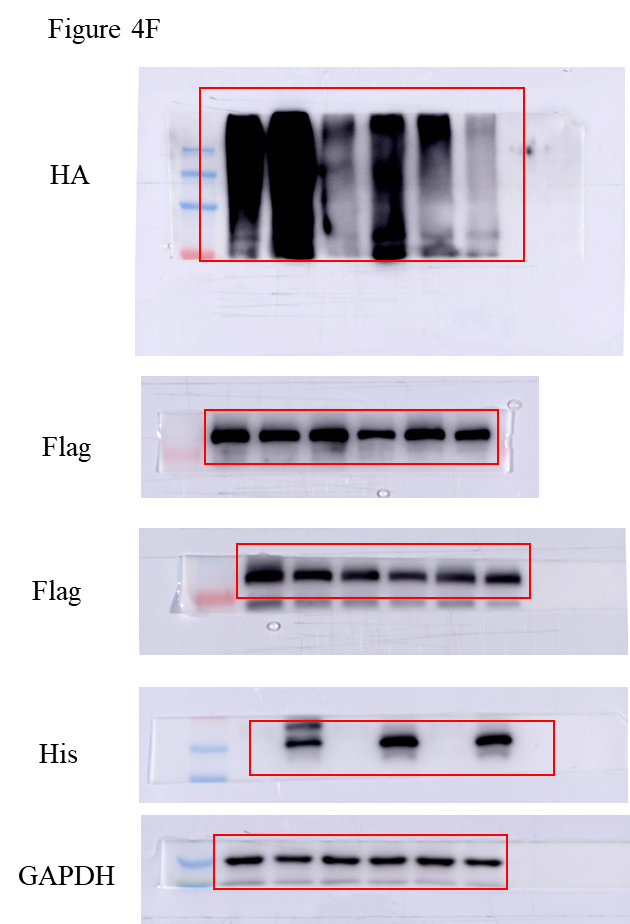


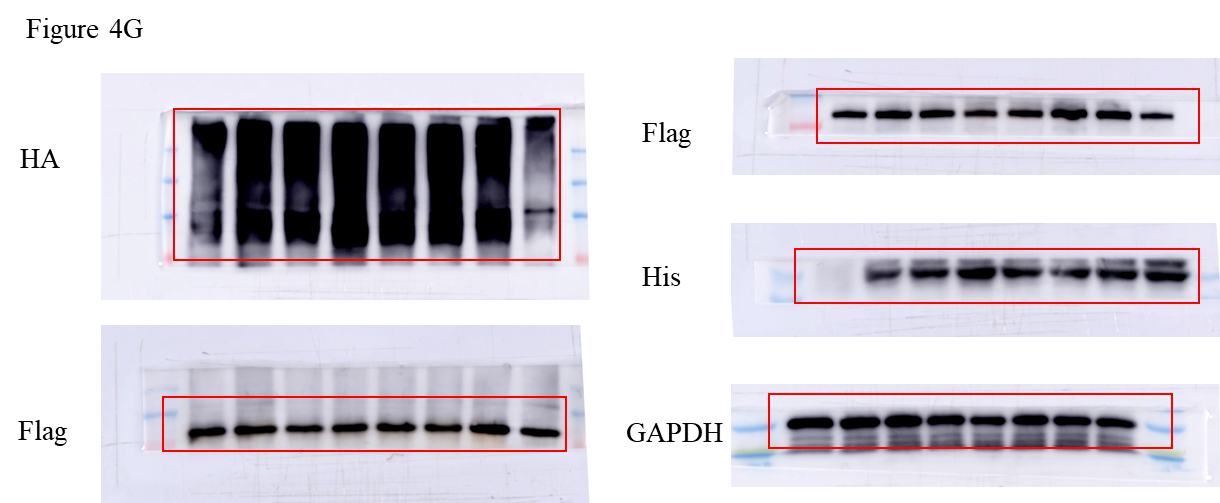


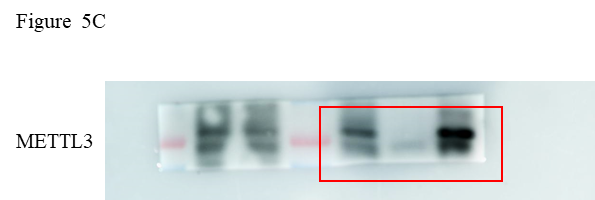

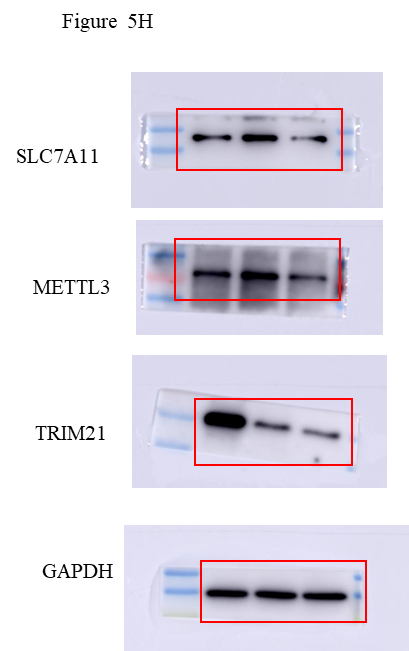

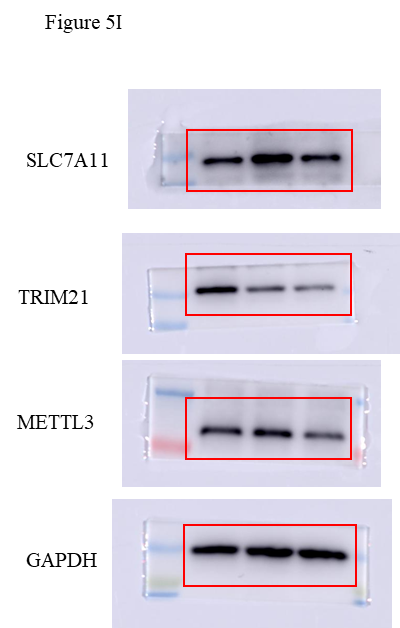


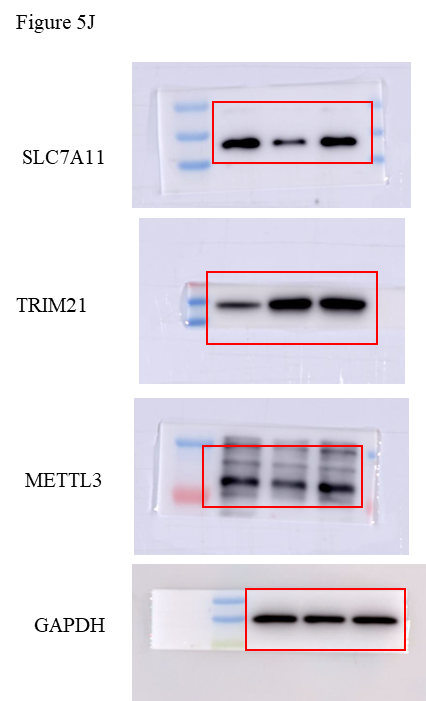

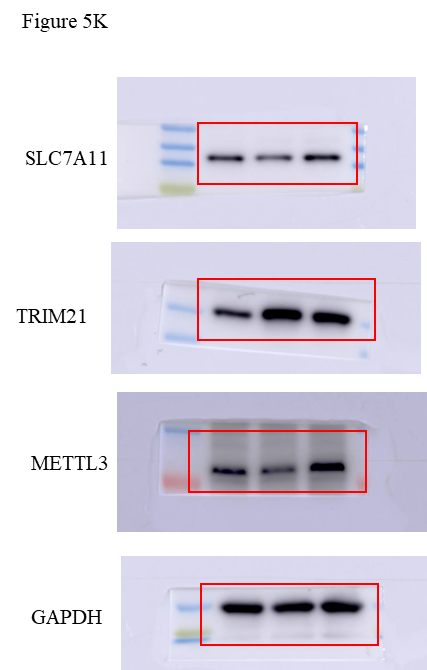


Supplementary materials

Full scans of uncropped blots presented in Supplementary Figures of the paper


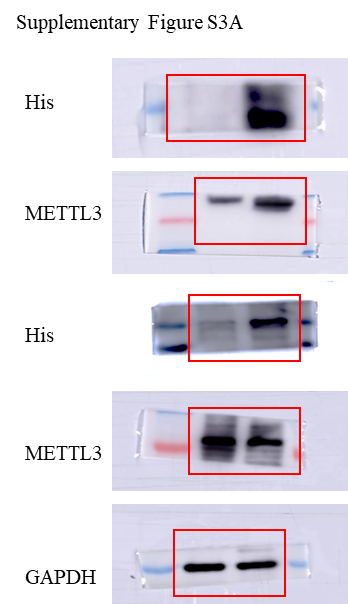

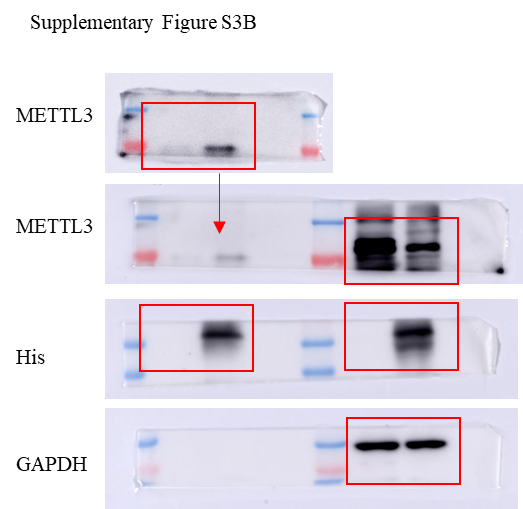

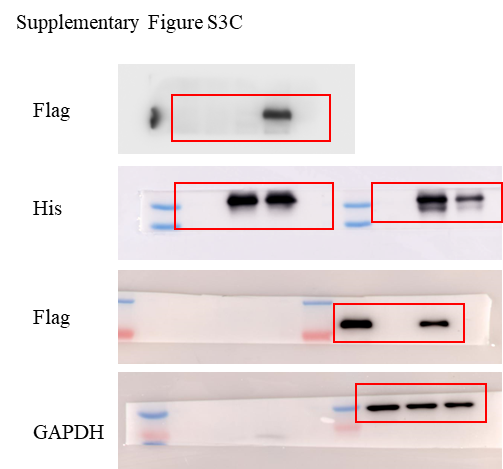

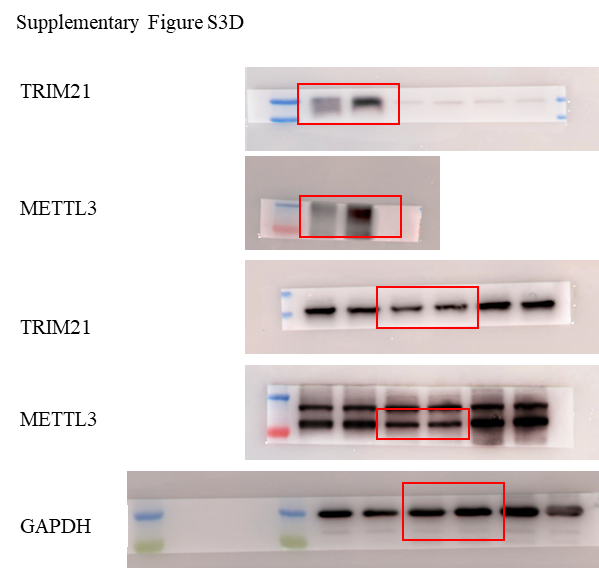

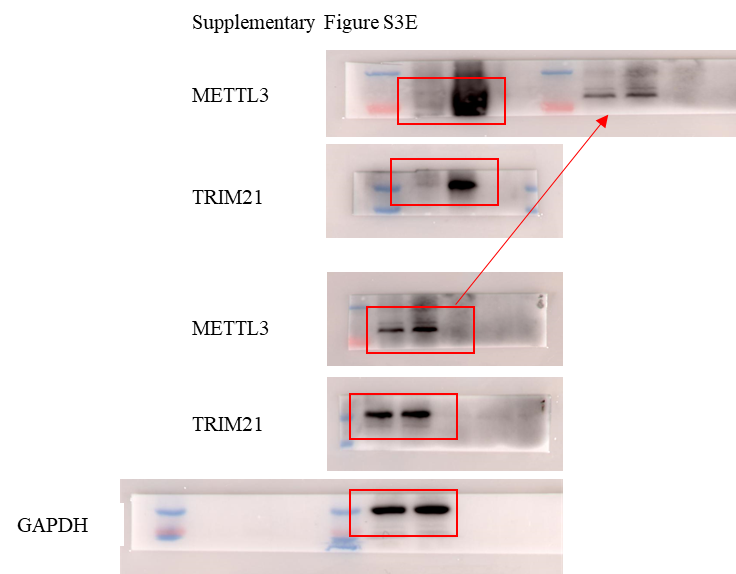

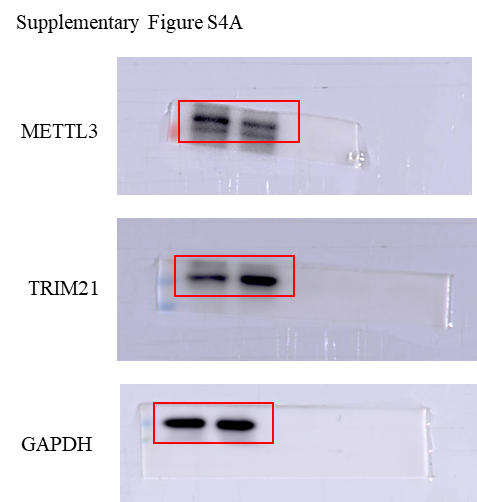

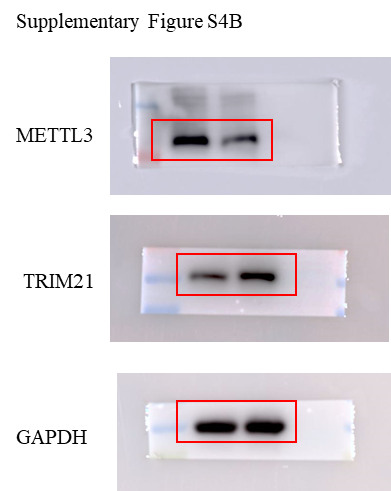

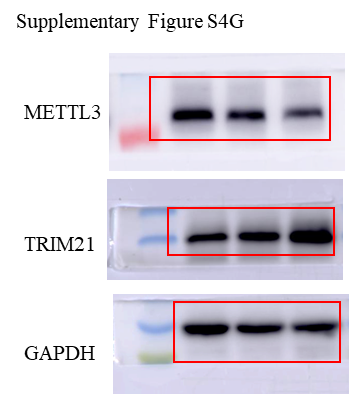

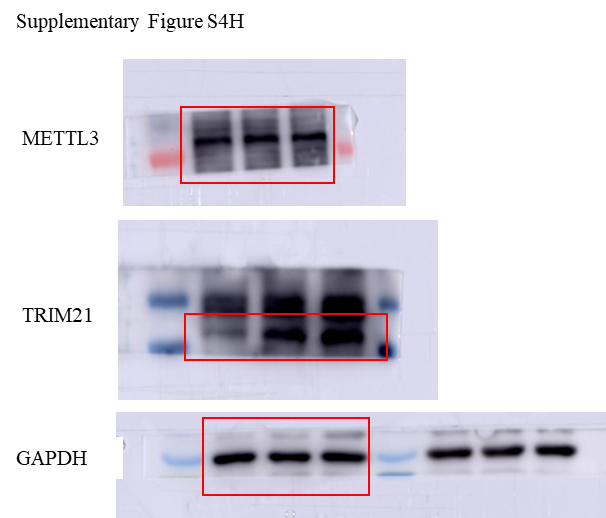

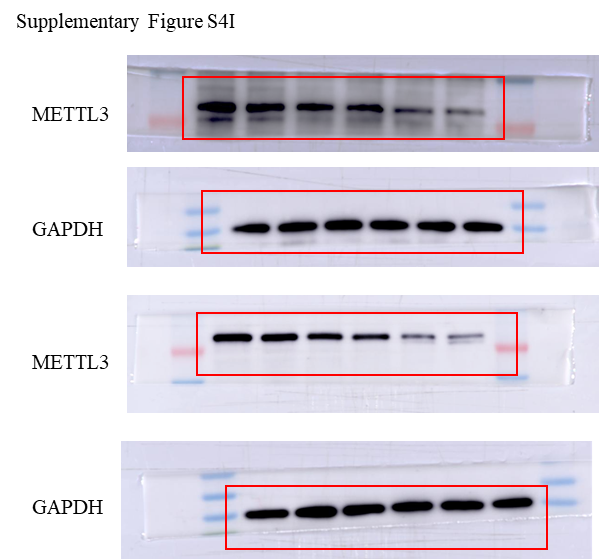


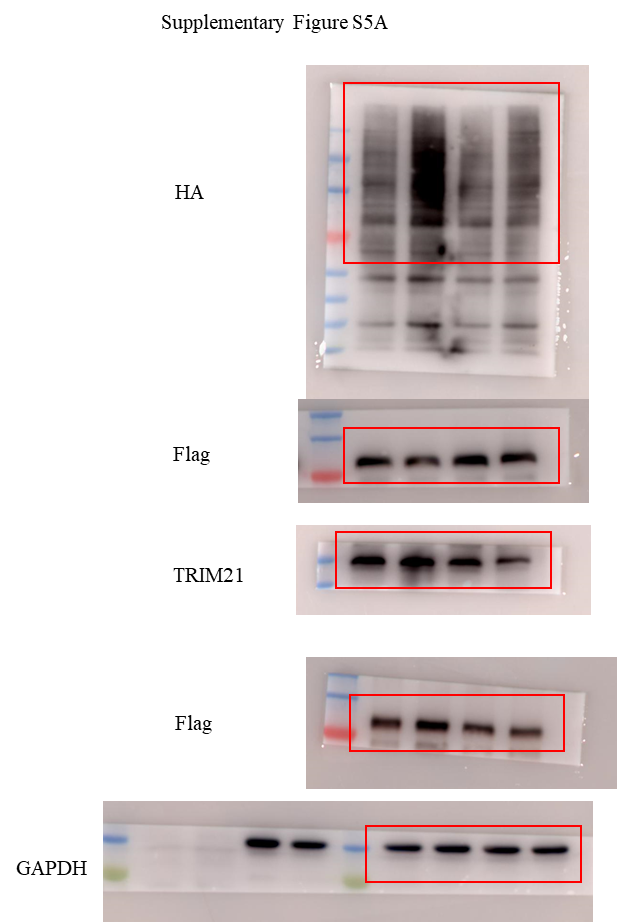

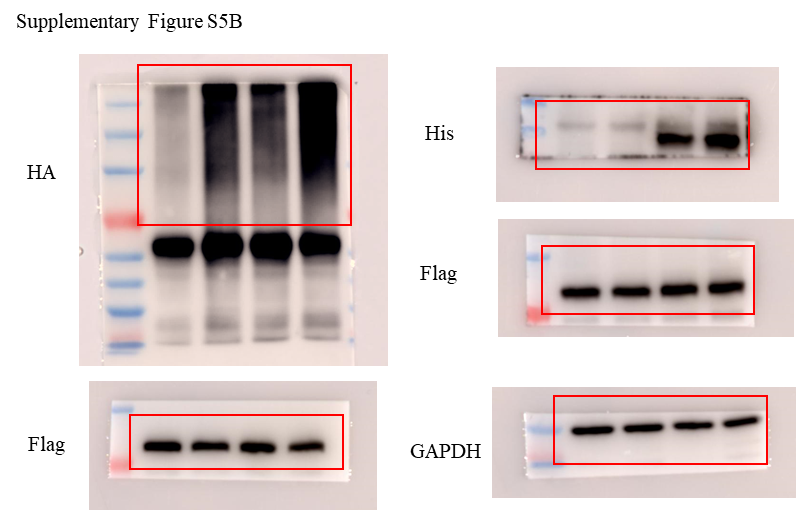


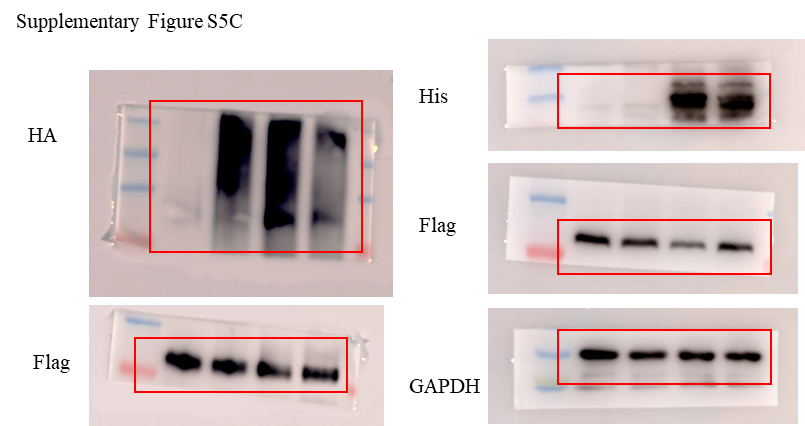


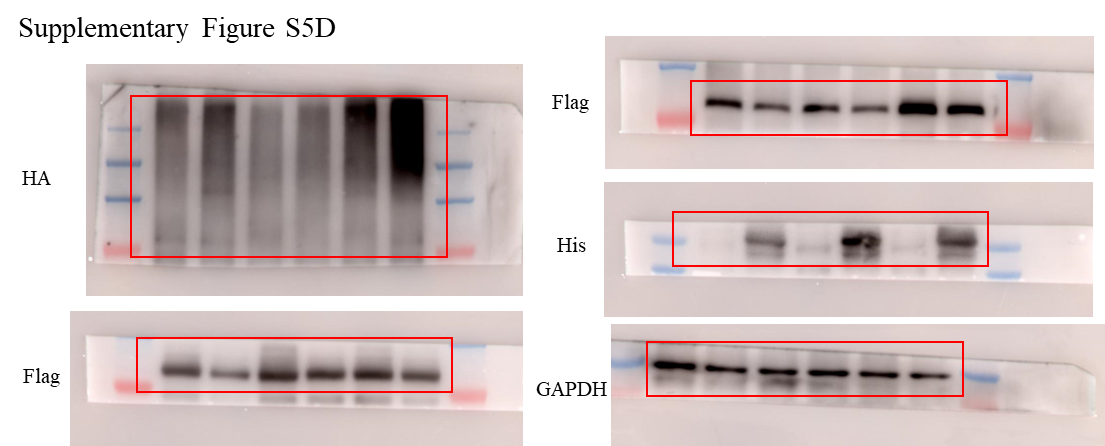


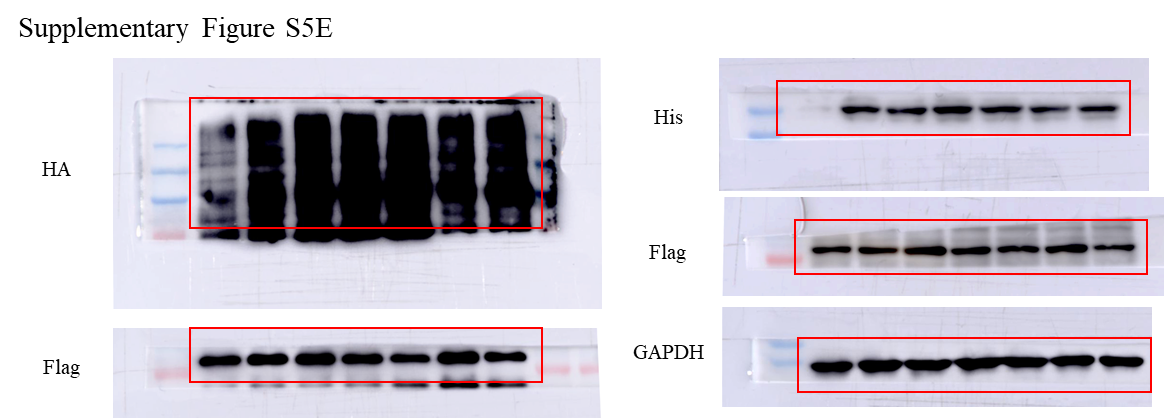


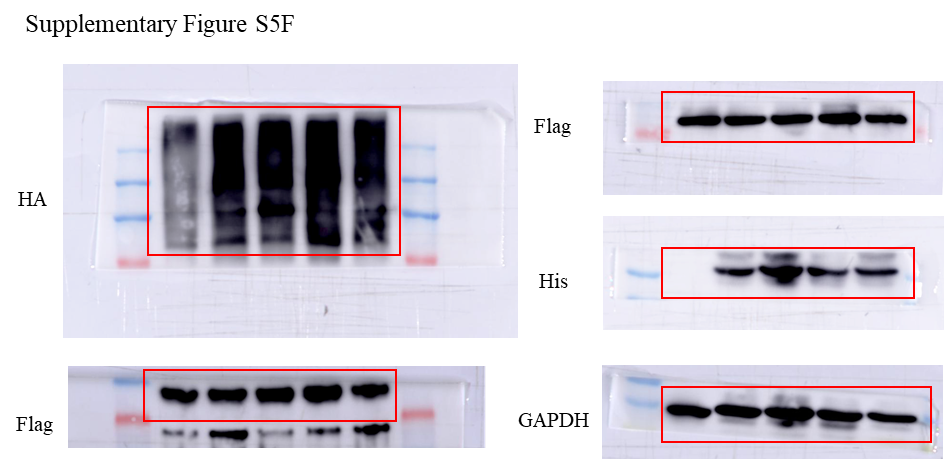


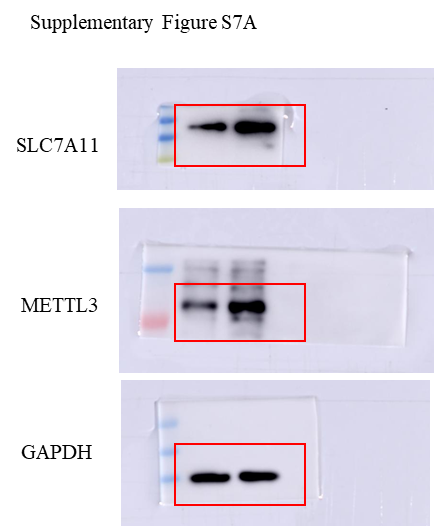

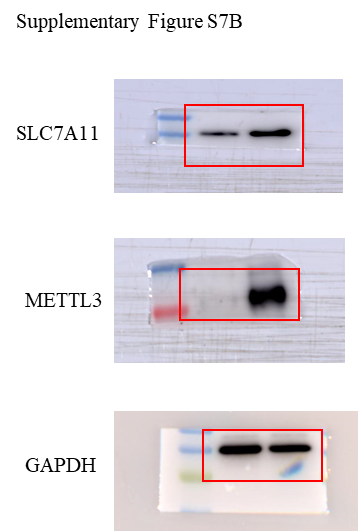


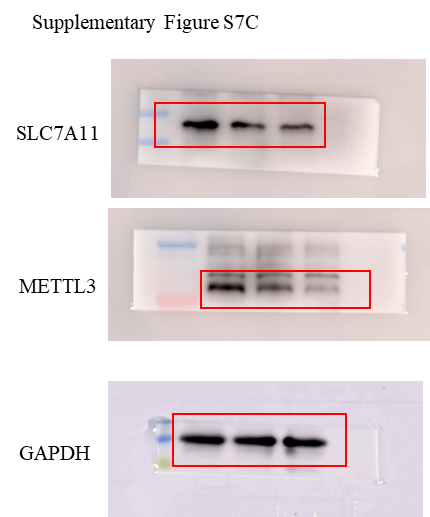

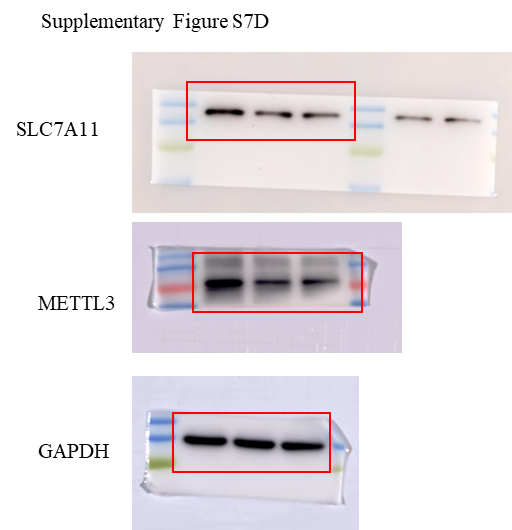

Supplement: Supplementary file 3 — Original western blots [file 41419_2025_7550_MOESM3_ESM.docx]
